# Valosin containing protein (VCP): initiator, modifier, and potential drug target for neurodegenerative diseases

**DOI:** 10.1186/s13024-023-00639-y

**Published:** 2023-08-07

**Authors:** Siwei Chu, Xinyi Xie, Carla Payan, Ursula Stochaj

**Affiliations:** 1 Department of Physiology, McGill University, Montreal, HG3 1Y6 Canada; 2 Quantitative Life Sciences Program, McGill University, Montreal, Canada

**Keywords:** Proteostasis, Neurodegeneration, Chaperone networks, Stress granules, Inclusion bodies, Valosin containing protein

## Abstract

The AAA^+^ ATPase valosin containing protein (VCP) is essential for cell and organ homeostasis, especially in cells of the nervous system. As part of a large network, VCP collaborates with many cofactors to ensure proteostasis under normal, stress, and disease conditions. A large number of mutations have revealed the importance of VCP for human health. In particular, VCP facilitates the dismantling of protein aggregates and the removal of dysfunctional organelles. These are critical events to prevent malfunction of the brain and other parts of the nervous system. In line with this idea, VCP mutants are linked to the onset and progression of neurodegeneration and other diseases. The intricate molecular mechanisms that connect *VCP* mutations to distinct brain pathologies continue to be uncovered. Emerging evidence supports the model that VCP controls cellular functions on multiple levels and in a cell type specific fashion. Accordingly, VCP mutants derail cellular homeostasis through several mechanisms that can instigate disease. Our review focuses on the association between VCP malfunction and neurodegeneration. We discuss the latest insights in the field, emphasize open questions, and speculate on the potential of VCP as a drug target for some of the most devastating forms of neurodegeneration.

**Supplementary Information:**

The online version contains supplementary material available at 10.1186/s13024-023-00639-y.

## Background

### Proteostasis and Protein Aggregation

Protein homeostasis, also known as proteostasis, is achieved through the coordination of protein synthesis, folding, posttranslational modification, and degradation. These activities require an intricate network of pathways and regulators that control proteostasis at the cell, organ, and organismal levels [1, 2].

Proteins fold in an environment with a high risk of inappropriate molecular interactions that promote aggregation [2, 3]. Molecular chaperones (here referred to as chaperones) and their co-chaperones cooperate to fold and maintain the functional state of individual proteins and higher order complexes; they also target proteins to degradation [2, 4]. Stress, the production of toxic proteins, or degradation overload can disrupt the proteostasis network [5]. The loss of proteostasis accelerates aging, compromises organismal health, and may culminate in cell death [2–4, 6]. Moreover, the derailment of proteostasis causes or aggravates human diseases and disorders [4, 7, 8]. Many neurodegenerative diseases are characterized by the aggregation of polypeptides and the formation of granules or inclusions [5, 6]. Pathological aggregates commonly form when misfolded proteins accumulate. While aggregates can be toxic, not all granules are harmful. Notably, ribonucleoprotein (RNP) assemblies are crucial hubs to regulate cellular homeostasis. Their organization and function are controlled by a process known as granulostasis [9]. As discussed below, valosin containing protein (VCP, also called p97 in mammals) is directly involved in numerous cellular activities that ensure proteostasis.

## Main text

### Valosin-containing protein, VCP

**Fig. 1:**
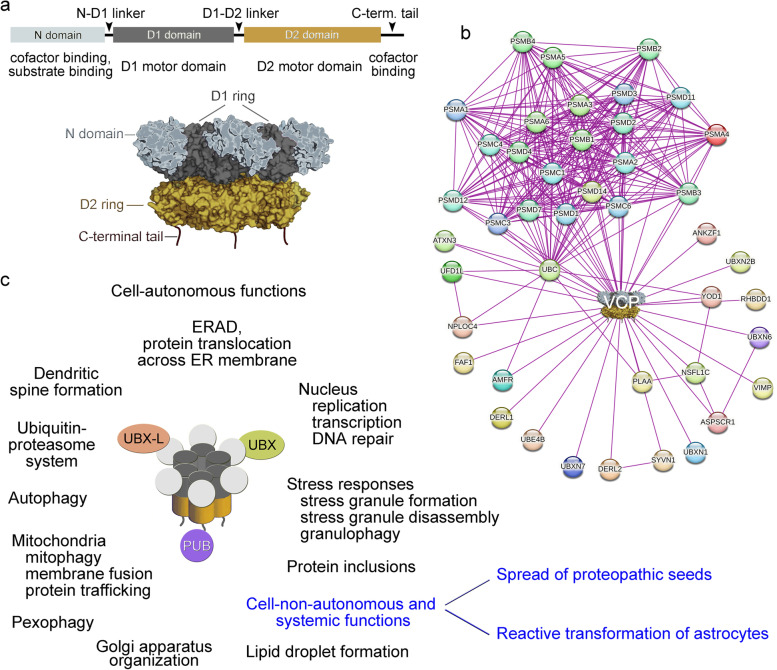
VCP protein organization and cellular interactions. **a** VCP domain organization and homohexamer formation. See text and [13] for details. **b** The STRING network of high confidence interactions (minimum score 0.70) of human VCP is depicted. The interactors are limited to the physical subnetwork [14]. The subnetwork includes many of the proteins that serve as VCP cofactors. **c** VCP-cofactor complexes (illustrated with cofactors that contain UBX-L, UBX, or PUB domains) regulate a vast number of cell-autonomous functions. *VCP* mutations may derail these activities. The cell-non-autonomous functions of VCP are beginning to emerge

The chaperone VCP is a type II ATPase associated with diverse cellular activities (AAA^+^ ATPase, Fig. 1a). In humans, the major product of *VCP* gene expression is a protein of 806 amino acid residues and apparent molecular mass of 90 kDa. VCP is evolutionarily conserved; homologs have been identified in yeasts (Cdc48), worms (CDC-48), and flies (TER94, transitional endoplasmic reticulum ATPase) [10]. VCP serves as an integral part of a larger network that is dedicated to establishing and preserving proteostasis [11, 12].

Work with *Saccharomyces cerevisiae* was instrumental to uncover the biology of VCP. In budding yeast, *CDC48* is an essential gene [15, 16]. Under non-permissive conditions, conditional mutants display diverse phenotypes [17–20]. They include cell cycle arrest, dysfunctional ubiquitin-dependent proteolysis, impaired ER membrane fusion and ERAD, abnormal cell morphology, aberrant spindles, aggregation of mitochondria, altered sensitivity to oxidative stress, and changes in the metabolome [21, 22]. By contrast, *CDC48* overexpression causes aberrant cell cycle progression in the G2 phase and enhances the toxicity of polyQ-expanded huntingtin polypeptides [23].

Mammalian VCP is an abundant chaperone which amounts to ~ 1% of the total protein in HEK293T cells. It promotes the extraction of ubiquitinated proteins from larger complexes for subsequent recycling or degradation. Furthermore, VCP contributes to additional processes that support cellular homeostasis and are often directly relevant to human health. To accomplish such diverse tasks, VCP associates with a wide variety of cofactors and other binding partners ([12, 24, 25], Fig. 1b, Table 1). Cofactors are defined as proteins with motifs or domains that directly bind the ATPase [26]. Cofactors with a ubiquitin-binding domain that recognizes ubiquitinated clients are classified as ubiquitin adaptors [27].

**Table 1:** Cellular functions associated with the ATPase VCP. Examples of cofactors that have been linked to specific cellular activities are listed. Components that are part of the UPS and autophagy network were collected from the BioGrid database [12]. Additional references are listed in the table. Most of the complex components interact with VCP directly. However, this has not always been established, and VCP association may be mediated by a protein that is part of a complex. Alternative protein identifiers used in original publications are shown in brackets

VCP-related functions	VCP complex component
ERAD	UFD1–NPL4, UBXD8, ATXN3, GP78/AMFR, SVIP, VIMP (SELS, SELENOS), SELK, UBX2 (SEL1), HRD1 (SYVN1, DERL3), DERL2, DERL1, NGLY1, UBXN1 (SAKS1), UBXN4 (UBXD2, erasin), RNF103 (KF1), TRIM13 (RFP2), UBXN6 (UBXD1), RHBDL4, RNF19A (Dorfin) [27–44]
Control of protein translocation into the ER	ZFAND2B (AIRAPL) [45]
Ubiquitin–proteasome system (UPS)	p47 (NSFL1C), RNF45, UFD1, ATXN3, NPL4, FAF1, VIMP, DERL1, FAF20, SYVN1, UBXN1. UBE4B, UBXN7, SPRTN, VCPIP1, YOD1, PLAA, SIK2, SVIP, UBC, PARK2, RNF31, BAG6, BRCA1, FBXW1, COPS5, Cul1, DERL2, UBQLN1, ZFAND2B, ANKRD13A, UBQLN2, USP13 [12]
General autophagy, mitophagy	UFD1–NPL4, PARK2, PINK1, MFN (mitofusin), OPTN (optineurin, FIP2), UBQLN2, USP13, UBXN1, WIPI2, WASHC4/SWIP [12, 46–49]
Mitochondria-associated degradation (MAD); mitochondrial protein translocation associated degradation (mitoTAD)	UFD1-NPL4, PLAA (UFD3/DOA1), UBXD8 (FAF2), ANKZF1 (VMS1), UBXD1 (UBXN6) [50–52]
Mitochondrial membrane fusion	MFN [50]
Mitochondria-ER contacts	VPS13D, UBXD8 [53, 54]
Protein trafficking; mitochondria → peroxisomes	MITOL (March5) [55]
Ribosome QC, ribophagy, damaged rRNA recognition	ANKZF1 (VMS1), UFD1-NPL4, UFD3, ribosome quality control complex (yeast: Rqc1p-Rkr1p-Tae2p-Cdc48p-Npl4p-Ufd1p) [12, 27, 56–58]
ER to Golgi trafficking; Golgi-ER membrane reassembly, ribosome-ER contacts	SVIP, p47 (NSFL1C), UFD1-NPL4, UBXN2B (p37), STX5A (syntaxin), VCIP135 [59–63]
Lysosome function and clearance, endolysosomal protein sorting	UBXD1 (UBXN6), PLAA, YOD1, SVIP [64–66]
Stress response, stress signaling, transcriptional stress	UFD1-NPL4, BAG1, MEST (mesoderm specific transcript), SMY2 (GIGYF1/2) [27, 67–72]
Stress granule assembly	UFD1L, PLAA [73]
Granulophagy	UBXD8 (UBX2), UFD1–NPL4 [27]
Stress granule disassembly	FAF2 (UBXD8, UBXN3B), ZFAND1 [74, 75]
Protein inclusions	RNF19A (Dorfin), TRIM21 [76, 77]
Replication	UFD1-NPL4-FAF1, TEX264 [78–80]
Transcription	RHBDL4, GP78 [43]
Accumulation of ubiquitinated proteins in nuclear blebs	UBXD1 (UBXN6) [81]
DNA damage repair	UFD1-NPL4, DOA1, MRE11-RAD50-NBS1, TEX264 [80, 82–86]
Dendritic spine formation	NF1 (neurofibromin), ATL1 [59, 87, 88]
Lipid droplet formation and turnover	UBXD8, SVIP [54, 89, 90]
Regeneration of free monoubiquitin; ubiquitin homeostasis	UFD1-NPL4 [91]
Ciliogenesis	UBXN10 (UBXD3) [11]
Apoptosis	UBXD8, SMY2 (GIGYF1/2) [54, 72]
Anti-viral immune responses	UFD1-NPL4, RIG-1/DDX58 [92]
Other processes	HIV disease progression [93], cancer biomarker [94, 95]

VCP assembles into a barrel-shaped homohexamer with six-fold radial symmetry [96–99]. The monomeric protein consists of an N-terminal domain (here called N-domain), followed by two ATPase domains (D1 and D2), and a short C-terminal tail ([13], Fig. 1a). The flexible N-terminal segment contributes to substrate selection by interacting with ubiquitin either directly or indirectly through cofactors [100]. The cofactors often bind ubiquitin with high affinity [101], which enhances the recruitment of substrates to VCP. The AAA^+^ motor domains D1 and D2 form a double-ring or barrel structure. The C-terminal tail also interacts with cofactors and contains a low complexity region ([101–105], Fig. 1a). Outside of the N-terminal and C-terminal VCP segments, the chaperone associates with non-canonical cofactors. For instance, neurofibromin 1 binds the ATPase domains of VCP [50]. VCP-cofactors form higher molecular complexes that are directly relevant to human health. Specific links between VCP-complexes have been established or predicted for neurodegeneration in general [106–108], amyotrophic lateral sclerosis (ALS [109–111]), Parkinson’s disease (PD [55, 112–115]), Alzheimer’s disease (AD [112, 116–119]), Huntington’s disease (HD [91, 120–122]) and others. Moreover, animal models support a role for VCP in fear memory and social behavior [59]. VCP affects additional health conditions that are not discussed here, such as HIV (disease progression [93, 123]) and cancer (disease biomarker [94, 95]).

### Biochemical properties of VCP

#### VCP ATPase activity

The chaperone function of VCP depends on its ATPase activity. Both ATPase domains of VCP (D1 and D2; Fig. 1a) include a Walker A and Walker B motif, which bind and hydrolyze ATP [30, 124]. ATP-binding to D1 promotes the assembly of VCP into its functional hexameric form [125]. As D1 has only low basal ATPase activity, the overall ATPase activity of VCP is provided by D2 [94, 126]. ATP hydrolysis drives major conformational changes in the D2-domain. It generates the force to segregate or unfold client proteins and their ubiquitin chains. Client unfolding and release are initiated by threading through the central opening of the VCP hexamer [94, 127–131]. On the other hand, distal ubiquitin chains may move through the lateral openings of the VCP hexamer [131]. Both the N-terminal domain and C-terminal tail modulate the VCP ATPase activity. This is achieved through cofactor binding and posttranslational modifications (PTMs) [127].

#### Subcellular distribution

VCP is present in different subcellular locations [132–134]. VCP’s functions are required in the nucleus and cytoplasm, and the ATPase shuttles between both compartments. A nuclear localization sequence (NLS) in the N-terminal domain of VCP (amino acid residues 60 to 66) promotes nuclear import [135]. Interestingly, the NLS is embedded in a region that binds various cofactors (see below), pointing to the possibility that cofactor interaction modulates VCP nuclear import. The C-terminal tail also impinges on the nucleocytoplasmic distribution of VCP [136], but the role of the C-terminal portion for nucleocytoplasmic transport is not fully understood. Nevertheless, the cell cycle-dependent phosphorylation of a tyrosine residue near the C-terminus correlates with the nuclear accumulation of yeast Cdc48 [137]. To our knowledge, no hydrophobic nuclear export signal recognized by Crm1 has been demarcated for VCP so far. VCP cysteine palmitoylation (Table 2) may support its association with membranes [127, 138].

**Table 2:**
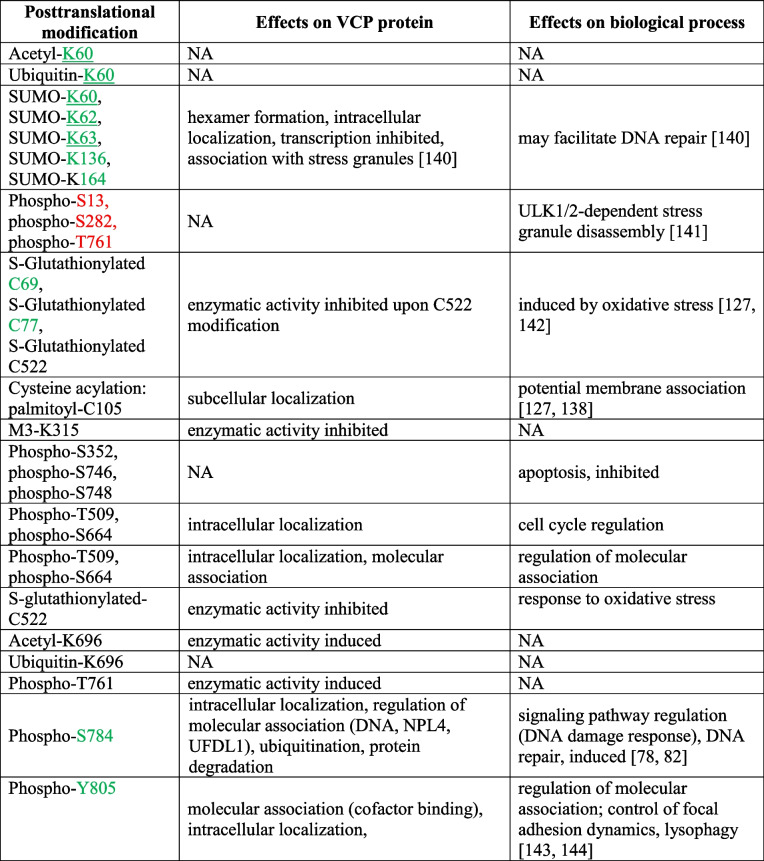
PTMs impact VCP-dependent biological activities. Information on VCP modifications was provided by PhosphoSitePlus [139] and the sources listed in the table. The table shows PTMs of residues located in regions that are relevant to the processes discussed in this review. PhosphoSitePlus [139] provides comprehensive information on all PTMs. Residues that are part of cofactor binding sites are in green. These sites are present in the N-terminal domain (residues 1–208; specifically Nn: 24–104; Nc: 113–184) and the C-terminal tail (residues 764–806). The nuclear localization sequence (NLS) encompasses residues 60 to 66 [135], underlined in the table. Cellular functions controlled by multiple PTMs are grouped together. Residues relevant to stress granule biogenesis are in red. M3, tri-methylation; phospho, phosphorylation; SUMO, sumoylation. NA denotes consequences of the PTMs that are not fully understood. See Fig. S1 for a comprehensive depiction of the PTMs that were identified for VCP

Reference: [78, 82, 127, 138, 140–144]

#### VCP posttranslational modifications (PTM)

VCP can be modified on multiple sites. Ubiquitination, acetylation, phosphorylation, palmitoylation, methylation, SUMOylation, and other modifications amount to at least 170 PTMs [127, 139]. A “combinatorial code” of PTMs [127] likely determines the functional consequences of a particular modification pattern. PTMs regulate the VCP ATPase activity, interactions with cofactors, client specificity, subcellular localization, and other parameters (Table 2, Fig. S1). For example, the VCP ATPase activity is increased upon phosphorylation of S770, but reduced by C522 S-glutathionylation [142, 145]. SUMOylation regulates critical aspects of VCP biology, such as hexamer formation, and the localization to nuclei or stress granules [140].

### The multifaceted contributions of VCP to cell biology

VCP is involved in numerous cellular processes, and the full spectrum of VCP-related functions continues to emerge [146]. Best characterized are VCP’s segregase activity and its role in the targeting of proteins to degradation. Both processes make fundamental contributions to cellular homeostasis. Especially relevant to neuronal health is the removal of aberrant proteins, organelles, and granular compartments [147, 148]. Cofactor(s) and the subcellular location of VCP-cofactor complexes determine the substrate specificity and consequences of VCP-dependent interactions. The following sections discuss in detail how VCP complexes contribute to specific cellular activities.

#### VCP protein complexes

**Fig. 2:**
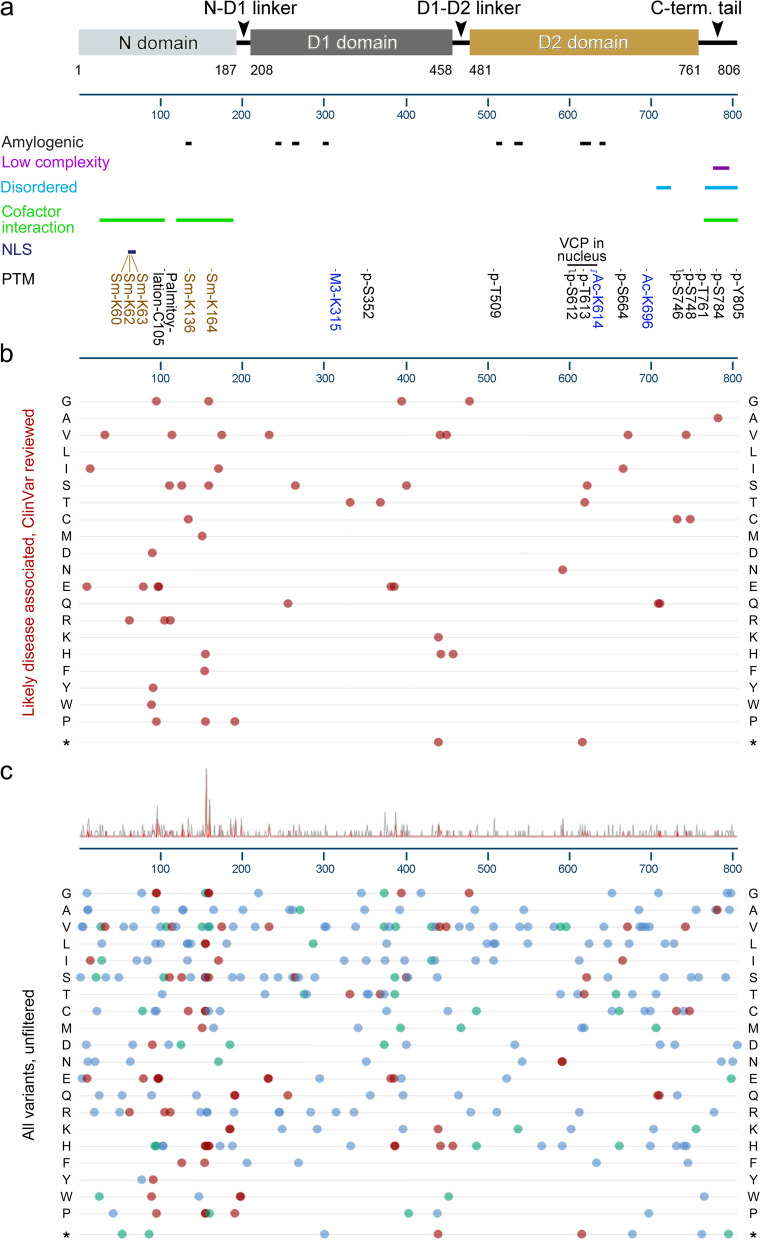
VCP regions, interaction sites, PTMs, and variants. **a** The domain organization, regions relevant to aggregation and interactions (amyloidogenic, low complexity, major sites for cofactor interaction), subcellular targeting (nuclear localization sequence, NLS), and PTMs with known impact on VCP localization or function are depicted. Sm, sumoylation; M3, tri-methylation; Ac, acetylation. **b** Likely disease-associated variants (neurodegeneration and other conditions) are depicted; they have been curated from ClinVar (NIH). The variants are linked to different diseases and disorders, such as neurodegeneration and cancer. **c** *VCP* variants linked to disease (red), with predicted consequences (blue), likely benign (light green), or with uncertain outcomes (dark green) are shown. The distribution of mutations appears on top of the table. **b**, **c** The variant amino acid residue is shown at the margins in the one letter code. * indicates nonsense mutations. The figure has been generated with information from multiple sources [135, 138, 139, 151, 152]

In most cases, VCP clients initially bind to cofactors which are linked to specific VCP activities (Table 1). Different VCP complexes have been connected to particular VCP functions in humans and rodents (Table S1). The properties and subcellular distribution of cofactors differ widely, and some cofactor-VCP interactions are cell type-specific [149, 150]. Nevertheless, a set of general features applies to these associations. The major contact sites in the N-domain (residues 1–208, Fig. 2) are located in two segments (Nn: 24–104; Nc: 113–184). Ubiquitin regulatory X (UBX)/UBX-L (UBX-like) domain, VCP-interacting motif (VIM), VBM (VCP-binding motif), or SHP (BS1, binding segment) promote binding to the N-domain [127]. Most cofactors interact with the N-terminal segment in a highly dynamic fashion [149]. A limited number of VCP cofactors associate with the C-terminal VCP tail (residues 764–806). PUB (PNGase/UBA or UBX containing proteins) and PUL (PLAP, Ufd3p, and Lub1p) domains facilitate these interactions [103–105, 127]. Cofactors bind VCP as monomers or heterodimers, as exemplified by the UFD1-NPL4 dimer.

Cofactors may inhibit or stimulate the ATPase activity of VCP; prominent examples are p47 (NSFL1C) and p37 (UBXN2B) [153]. Notably, the response to p47 and p37 binding is dysregulated for disease-relevant VCP mutants [153].

VCP hexamers can associate with different cofactors at the same time. However, some cofactor combinations are mutually exclusive [127]. As such, binding of UBX domains (examples: UBXN7, UBXN8) will preclude the interaction with VIM motifs (present in AMFR and SELENOS) [154]. Table S2 provides more detailed information on major VCP binding partners, including the relevant pathways, protein and transcript abundance.

Pharmacological VCP ATPase inhibitors can strengthen or reduce the cofactor interactions. However, this does not apply to all cofactors, as some bind independently of VCP activity [149]. Interestingly, the association with VCP can determine cofactor stability and thereby the cellular activities that rely on these cofactors.

#### Protein degradation, organelle and granule removal

Proteolysis limits the harmful accumulation of aggregated proteins [2]. The ubiquitin–proteasome system (UPS) and the autophagy-lysosomal pathway (ALP) are the major routes of intracellular protein degradation [155]. UPS efficiently degrades individual proteins, while ALP eliminates large protein complexes and organelles [156].

Proteasomes recognize and degrade ubiquitinated clients in the cytoplasm and nucleus [157]. They also participate in endoplasmic reticulum associated degradation (ERAD) [155], ribosome-associated protein quality control (RQC) [56], and mitochondria-associated degradation (MAD) [50].

In mammalian cells, macroautophagy, microautophagy, and chaperone-mediated autophagy conclude with lysosomal degradation [155]. Dysfunctional organelles are removed by selective autophagy, which eliminates defective mitochondria (mitophagy), lysosomes (lysophagy), ER (reticulophagy), peroxisomes (pexophagy), and portions of the nucleus (nucleophagy) [158]. Granulophagy, another specialized form of autophagy, clears non-membrane bound compartments, such as stress granules [159].

VCP controls protein degradation on multiple levels [107, 141, 158, 160, 161]. First, in cooperation with cofactors, VCP targets ubiquitinated clients to the proteasome. Second, VCP controls several steps of autophagy to ensure the proper balance between repair and removal of damaged organelles [158, 162–164]. Third, VCP regulates granulostasis, thereby preventing the formation and dispersal of permanent protein aggregates. This is relevant to the nervous system, as lysophagy is triggered by neurotoxic aggregates to limit their spread in vitro [159, 162].

The dynamic interplay between VCP and other components of the proteostasis network adds further complexity to the control of proteostasis. For example, the E3 ubiquitin protein ligase CHIP and VCP bind mutant superoxide dismutase 1 (SOD1_G93A_) [165]; both regulate SOD1_G93A_ proteolysis. SOD1_G93A_ degradation relies on the collaboration of CHIP, hsp70, the hsp70 co-chaperone Bag-1, S6/S6’ (AAA^+^ ATPases in the 19S regulatory subunit of the proteasome), and VCP [165]. Bag-1 enables the formation of a ternary CHIP/Bag-1/VCP complex [165]. This may involve two Bag-1 binding sites in the D1 domain of VCP [70]. The importance of the Bag-1/VCP interaction goes beyond the removal of SOD1_G93A_. Bag-1 also regulates ERAD, at least for some VCP clients [70]. Taken together, CHIP and Bag-1 illustrate the intricate connections between VCP and other pillars of the intracellular proteostasis network. The communication among these factors may facilitate alternative routes to protein degradation and serve as a safety net to handle misfolded clients.

#### ER, ERAD

VCP controls ER morphogenesis [88] and ERAD [166]. Several VCP binding proteins, such as p47 (NSFL1C) and Atlastin-1, are implicated in the biogenesis of the ER network [167]. The knockdown of *VCP* or cofactor genes and the overexpression of pathogenic *VCP* mutants derail ER homeostasis in the nervous system [59, 88, 168]. These conditions reduce the extension of the ER into dendrites, both in cultured neurons and in mouse brains. Ultimately, this impairs proper dendritic spine formation.

During ERAD, VCP associates with ubiquitin ligases located at the ER [169] and extracts misfolded proteins, commonly with the assistance of UFD1-NPL4 (Table 1). The association of VCP with the ER is, at least in part, supported by gp78 [170]. The trafficking control of GABA_A_ receptors and the myelination of axons demonstrate the importance of ERAD in the nervous system [171].

#### Mitochondrial membrane fusion, mitophagy, mitochondria-associated degradation

Mitochondria control several branches of cell metabolism, calcium homeostasis, and cell intrinsic routes of apoptosis [172]. Mitochondrial homeostasis depends on fusion and fission, import of nuclear-encoded proteins from the cytoplasm, and removal of dysfunctional organelles. VCP regulates mitochondrial performance on multiple levels. The ATPase modulates (i) mitochondrial fusion, (ii) mitophagy, (iii) mitochondria-associated degradation (MAD), including mitochondrial protein translocation associated degradation (mitoTAD), (iv) mitochondrial calcium uptake, and (v) cell death [46, 50, 56, 173]. VCP affects these events through ubiquitin-dependent and ubiquitin-independent activities.

Mitochondrial abnormalities linked to mutant VCP include elevated reactive oxygen species (ROS) levels, mitochondrial uncoupling, reduced ATP production, mitochondrial fusion defects, and impaired clearance of damaged mitochondria [50, 114, 173, 174]. Loss of mitochondrial quality accompanies aging and neurodegenerative disorders, such as ALS, PD, and HD [172].

#### Ribosome-associated protein quality control (RQC)

Quality control of de novo synthesized proteins is needed to maintain a functional proteome [175, 176]. (The quality control of *ribosome biogenesis* is not discussed here; the subject has been reviewed recently [177].) RQC takes place at every step of translation; it regulates translation initiation, elongation, termination, and the recycling of ribosomal subunits [175]. Major tasks of RQC are the resolution of stalled or collided ribosomes and the removal of aberrant nascent peptides. For RQC-mediated nascent polypeptide degradation, VCP and other key regulators are recruited to the 60S ribosomal subunit [56, 57]. UFD1-NPL4 and ANKZF1 (VMS1) are major VCP cofactors that participate in RQC [56, 57]. For instance, ANKZF1 (VMS1) promotes the release of nascent peptide chains from the 60S subunit [178]. Subsequently, VCP in complex with other factors stimulates the removal of the defective translation products [179]. RQC is crucial to maintain the health of the nervous system, and impaired RQC is associated with different forms of neurodegeneration [57, 175, 180].

#### Golgi apparatus, vesicular trafficking, lysosomes

VCP promotes the reassembly of the Golgi apparatus after mitosis [61, 62]. The ATPase is also involved in protein trafficking from the ER to the Golgi apparatus [60], endolysosomal sorting of ubiquitinated proteins [64, 162, 181], and the elimination of dysfunctional lysosomes [64–66].

Due to their roles in autophagy and cell signaling, lysosomes serve as control hubs for cellular homeostasis [182]. Impaired performance of the autophagy-lysosomal pathway (ALP) is a major contributor to neurodegenerative diseases, including FTD, ALS, PD, and AD [182, 183]. Interestingly, neurodegeneration can arise from lysosomal dysfunction in neurons as well as non-neuronal cells, such as astrocytes and microglia [182].

Several VCP activities ensure the proper execution of ALP. For example, VCP conducts the endo-lysosomal damage response, called ELDR [65]. In collaboration with the ELDR components UBXD1, PLAA (phospholipase A2-activating protein, Doa1), and YOD1 (deubiquitinase), VCP stimulates the removal of damaged lysosomes. During ELDR, VCP and the ELDR components are recruited to damaged lysosomes. Upon recruitment, at least a portion of UBXD1, PLAA, and YOD1 colocalize with VCP on dysfunctional lysosomes.

Impaired ELDR is associated with several VCP variants that cause multisystem proteinopathy 1 (MSP1, formerly referred to as IBMPFD). Lysosomal clearance is compromised in skeletal muscle tissue from patients producing VCP_R155H_ or VCP_R93C_ variants. In mouse or cell culture models, VCP_R155H_, VCP_L198W_, and VCP_A232E_ reduce autophagic flux and clearance [65]. Earlier studies, using cell culture, mouse models, and patient samples, uncovered that VCP_R155H_, VCP_A232E_ and VCP_E578Q_ regulate autophagosome maturation, autophagic flux, and autolysosome formation in skeletal muscle and cultured cells [184].

The importance of ALP in human neurons is illustrated by the VCP cofactor PLAA, which controls endolysosomal proteostasis at the synapse [185]. Especially, PLAA directs (i) post-endocytic trafficking of signaling receptors required for neural development and (ii) the ubiquitin-dependent sorting of synaptic vesicle factors during recycling.

Taken together, alterations in VCP and its cofactors can derail ALP in skeletal muscle, neurons, and non-neuronal cells of the nervous system. Cellular and animal models as well as patient-derived samples have linked these changes to lysosomal damage and dysfunction [65, 184–186]. Several VCP variants that compromise ALP are established causes of MSP1.

#### Nucleus, replication, cell cycle progression, DNA damage response, transcription

VCP shuttles between the nucleus and the cytoplasm. This dynamic localization is important for the control of nucleophagy, nuclear size [187], replication [78, 79, 188], cell cycle progression [135, 189], intranuclear quality control/splicing [190–193], genome integrity [194], and the response to transcriptional stress or DNA damage [72, 82, 84, 85, 195]. VCP mutants with aberrant nucleocytoplasmic distribution contribute to the pathology of MSP1, ALS, hereditary spastic paraplegia (HSP), and PD ([136], see below).

The broad impact of VCP-dependent activities in the nucleus is exemplified by the removal of SUMOylated and ubiquitinated proteins at the replication fork [78] and mitotic spindle formation [189]. It is also relevant to lipid droplet assembly in the cytoplasm ([43], see below). The kinase Aurora A is necessary to form mitotic spindles [196], but VCP restricts the association of Aurora A with centrosomes [197]. To dismantle mitotic spindles, VCP removes Aurora A and other spindle assembly factors from chromatin [196, 198]. *VCP* overexpression stimulates the degradation of Aurora A, whereas VCP inhibition increases the abundance of Aurora A [189]. The control of Aurora A levels is driven by the collaboration between VCP and ER membrane protein complex subunit 3 (EMC3) [189]. Together, VCP and its binding partner EMC3 control the cell cycle by orchestrating the progression of M phase.

VCP regulates additional nuclear events that ensue in a wide-reaching control of organismal homeostasis. This is illustrated by the accumulation of fat and lipid droplets (LDs) in experimental rodents on a high fat diet [43]. Studies in mice and HepG2 cells (hepatocellular carcinoma) uncovered the underlying mechanism as changes in the activation of the transcription factor sterol regulatory element binding factor 1 (SREBP1). In the nucleus, SREBP1 controls the transcription of target genes involved in lipid biosynthesis. To enter the nucleus, SREBP1 has to be cleaved in the ER membrane. SREBP1 proteolytic processing relies on the collaboration of ubiquitinated SREBP1, VCP, and the rhomboid protease RHBDL4 [43]. Thus, *VCP* knockdown or pharmacological VCP inhibition (NMS-873) reduces SREBP1 cleavage. The mutation VCP_A232E_ compromises the interaction with RHBDL4 and ubiquitinated SREBP1. When kept on a high fat diet, *VCP*^*A232/*+^ knock-in mice accumulate less fat in the liver, have a larger intra-abdominal fat mass, and improved insulin tolerance.

In summary, the distribution of VCP between the nucleus and the cytoplasm is dynamic. Both cytoplasmic and nuclear VCP control functions that are located in the nucleus.

#### Ciliogenesis

Non-motile primary cilia control signaling in a wide variety of cell types [199, 200]. Primary cilia regulate the metabolism and cell migration in neurons and the metabolism in astrocytes [199, 201, 202]. Moreover, primary cilia of adult neural stem cells are essential for adult neurogenesis. On the other hand, dysfunctional primary cilia shape the pathological signaling events associated with ALS, PD, and AD [203]; they also instigate retinal diseases [204]. The proteome of primary cilia includes VCP, several interacting proteins and cofactors, such as SQSTM1/p62, NSFL1C, VCPIP1, optineurin (OPTN), and ANKRD13A [205].

The proper biogenesis of primary cilia and ciliary signaling depend on VCP [205]. These processes require the ciliary localization of VCP and some of its cofactors [11, 205]. For instance, VCP and UBXN10 are indispensable for ciliogenesis. Together, VCP and UBXN10 control the anterograde macromolecular transport into cilia [11]. Interestingly, a bidirectional link connects ciliopathies to autophagy [206]; this interplay could involve members of the VCP protein network.

#### Cell survival and death

VCP controls cell viability, but is also closely associated with the regulation of cell death. Mechanistic links between VCP and cell death are underscored by VCP inhibitors that trigger cancer cell death [72, 95, 207–209]. VCP-related death involves mitochondrial pathways, ER homeostasis, or autophagy.

VCP cofactors regulate the trajectory towards cell survival or death; they can have anti- or pro-apoptotic activities [54, 72]. For example, the adaptor UBXD8 recruits VCP to mitochondria and promotes the degradation of the pro-apoptotic factors Noxa, Bik, and Bnip3 [54]. In this scenario, the UBXD8-dependent degradation of Noxa restricts apoptosis [54].

The cofactor SVIP controls ERAD and autophagy by inhibiting VCP [210, 211]. SVIP abundance is developmentally regulated in an organ-specific fashion. Prolonged and excessive ER stress can increase the abundance of SVIP and ultimately cause apoptosis, at least in some cell types [210]. Derlin-1 (DERL1), another component of VCP complexes involved in ERAD, also facilitates cell death following extensive ER stress [212].

Taken together, the developmental stage, physiological conditions, and cell type determine the role of VCP in cell fate decisions. VCP cofactors shape these decisions.

#### Granulostasis

Eukaryotic cells contain diverse ribonucleoprotein (RNP) complexes that often organize into complex RNA granules. These granules regulate RNA metabolism and thereby impact cellular homeostasis [9]. Neuronal RNP granules move mRNA from the soma to cell processes for localized translation. Such localized protein synthesis controls neuronal plasticity and is essential to learning and memory [213]. Fear memory has been connected to VCP [59].

Many RNA granules are produced constitutively, while others form under special conditions, such as acute or chronic stress [214, 215]. Stress granules (SGs) assemble when oxidants, heat, viral infection, or other adverse events interfere with translation initiation [216]. Transient SGs formed during acute stress are mostly cytoprotective. By contrast, chronic stress generates cellular inclusions that differ in composition from their acute stress counterparts. Chronic SGs are less dynamic, resistant to disassembly, and may promote cell death [215]. Neurodegeneration is associated with elevated ROS levels and chronic inflammation [217]; both contribute to the accumulation of permanent inclusions.

The molecular mechanisms maintaining granulostasis are essential for neuronal health. In particular, the malfunction of individual granulostasis factors can prompt or accelerate the decline of CNS or PNS performance. Studies in different model systems established that VCP serves as a critical granulostasis factor (summarized in Table 3). Many of the insights described here relate to acute stress, although chronic is most relevant to neurodegeneration. Nevertheless, transient SGs can convert to persistent structures, and both granule types have common features. Thus, the knowledge generated with acute SGs is pertinent to the inclusions observed during neurodegeneration.

**Table 3:** The role of VCP in granulostasis. All comparisons are between controls and the experimental condition listed in the table. Abbreviations: acute, acute stress; CB-5083, reversible competitive inhibitor of VCP [142]; *Δ,* gene deletion; Eeyarestatin 1, directly binds VCP, preferentially interacts with membrane-associated VCP, inhibits ERAD [218]; MG132, proteasome inhibitor; NA, not applicable; NMS-873, allosteric VCP inhibitor [149]. Cellular models: HEK293, human embryonic kidney epithelial cells; HeLa, human cervix adenocarcinoma, epithelial cells; MEF, mouse embryonic fibroblast; Neuro2a, mouse neuroblastoma cells; U2OS, human osteosarcoma cells, epithelial morphology; *S. cerevisiae,* budding yeast

Experimental condition	SG inducer	Phenotype related to SGs	Model system	Reference
*VCP* knockdown	heat, acute	impaired SG clearance	HeLa	[159]
*VCP* knockdown	heat, acute	impaired SG clearance	U2OS	[141]
*VCP* knockdown	arsenite, acute	impaired SG formation	HeLa	[73]
*VCP* knockdown	MG132, acute	impaired SG formation	HeLa	[73]
VCP pharmacological inhibition	heat, acute	impaired SG clearance	HeLa	[159]
VCP pharmacological inhibition	arsenite, acute	impaired SG formation	HeLa	[73]
VCP pharmacological inhibition	MG132, acute	impaired SG formation	HeLa	[73]
VCP pharmacological inhibition (Eeyarestatin 1)	heat, acute	impaired SG clearance	U2OS	[141]
VCP pharmacological inhibition (CB-5083)	heat, acute	impaired SG clearance	HeLa	[219]
VCP pharmacological inhibition (CB-5083)	arsenite, acute	impaired SG clearance	HeLa	[219]
VCP pharmacological inhibition (NMS-873)	heat, acute	impaired SG clearance	HeLa	[219]
VCP pharmacological inhibition (NMS-873)	arsenite, acute	impaired SG clearance	HeLa	[219]
VCP pharmacological inhibition (CB-5083)	heat, acute	impaired SG clearance	U2OS	[68]
*VCP*_*R155H*_ knock-in	NA	increased levels of proteins with oxidative damage; levels of G3BP1, eIF2α, p-eIF2α unchanged	*VCP*_*R155H*_ knock-in mouse	[67]
*VCP*_*R155C*_ overexpression	arsenite, acute	reduced localization of VCP in SGs and nucleus	HEK293	[140]
*VCP*_*R155H*_ overexpression	NA	constitutive SGs; SGs contain eIF3 subunits, TDP-43, VCP	HeLa	[159]
*VCP*_*R155H*_ overexpression	heat, acute	no SG clearance defect	C2C12	[67]
*VCP*_*R155H*_ overexpression	heat, acute	impaired SG clearance	U2OS	[74]
*VCP*_*R155H*_ overexpression	arsenite, acute	impaired SG clearance	C2C12	[67]
*VCP*_*R159H*_ overexpression	arsenite, acute	reduced localization of VCP in SGs and nucleus	HEK293	[140]
*VCP*_*A232E*_ overexpression	arsenite, acute	impaired SG clearance	C2C12	[67]
*VCP*_*A232E*_ overexpression	heat, acute	no SG clearance defect	C2C12	[67]
*VCP*_*A232E*_ overexpression	heat, acute	impaired SG clearance	U2OS	[141]
*VCP*_*A232E*_ overexpression	NA	constitutive SGs; SGs contain eIF3 subunits, TDP-43, VCP	HeLa	[159]
*VCP*_*A232E*_ overexpression	heat, acute	impaired SG clearance	U2OS	[74]
*VCP* wild type overexpression	MG132	VCP and Dorfin colocalize in aggresome	HEK293	[76]
*VCP*_*K524A*_ co-expression with SOD1_G85R_	NA	reduced Dorfin-dependent ubiquitination of SOD1_G85R;_ VCP_K524A_ does not prevent binding of Dorfin to SOD1_G85R_	HEK293, Neuro2a	[76]
*UFDL1* knockdown	arsenite, acute	impaired SG formation	HeLa	[73]
*UFDL1* knockdown	MG132, acute	impaired SG formation	HeLa	[73]
*PLAA* knockdown	arsenite, acute	impaired SG formation	HeLa	[73]
*PLAA* knockdown	MG132, acute	impaired SG formation	HeLa	[73]
*ZFAND1* knockdown	heat, acute	SG clearance not impaired, VCP recruitment to SGs not reduced	HeLa	[75]
*ZFAND1* knockdown	arsenite, acute	impaired SG clearance, reduced VCP recruitment to SGs	HeLa	[75]
*ZFAND1* knockdown	hydrogen peroxide, acute	SG clearance not impaired, VCP recruitment to SGs not reduced	HeLa	[75]
*ZFAND1* knockdown	osmotic stress, acute	SG clearance not impaired, VCP recruitment to SGs not reduced	HeLa	[75]
Control; wild type *VCP,* endogenous levels	heat, acute	VCP-ULK1/2 binding increased; VCP recruited to SGs; ULK1/2 located in SGs	MEF	[141]
*ULK1/2* knockdown	arsenite, acute	impaired SG clearance	MEF	[141]
*ULK1/2* knockdown	heat, acute	impaired SG clearance	MEF	[141]
*ULK1/2* knockdown plus phosphomimetic VCP mutant	heat, acute	SG clearance rescued	MEF	[141]
ULK1/2 pharmacological inhibition	heat, acute	impaired SG clearance	U2OS	[141]
ULK1/2 pharmacological inhibition	arsenite, acute	impaired SG clearance	U2OS	[141]
ULK1/2 pharmacological inhibition	heat, acute	impaired SG clearance	C2C12	[141]
ULK1/2 pharmacological inhibition	arsenite, acute	impaired SG clearance	C2C12	[141]
ULK1/2 agonist	heat, acute	faster SG clearance	U2OS	[141]
*VCP* knockdown plus ULK1/2 agonist	heat, acute	impaired SG clearance	U2OS	[141]
*FAF2 (OPTN)* knockdown	heat, acute	impaired SG clearance	U2OS	[74]
*G3BP1/2* double knockdown plus ubiquitination deficient G3BP1 mutants	heat, acute	reduced VCP recruitment to SGs	U2OS	[74]
*ubx2Δ*	NA	SG accumulation	*S. cerevisiae*	[159]
*vms1Δ*	NA	SG accumulation	*S. cerevisiae*	[159]
*Cdc48* temperature sensitive allele; non-permissive temperature	NA	SG accumulation	*S. cerevisiae*	[159]
*Ufd1* temperature sensitive allele; non-permissive temperature	NA	SG accumulation	*S. cerevisiae*	[159]
*Npl4* temperature sensitive allele; non-permissive temperature	NA	SG accumulation	*S. cerevisiae*	[159]

##### Genetic and biochemical evidence links VCP to granulostasis

Stress generally elicits the ubiquitination of proteins; the precise patterns of ubiquitination are determined by the type of stress. VCP-cofactor complexes recognize ubiquitinated substrates, including those present in SGs and disease-related inclusions. VCP is an evolutionarily conserved protein that modulates granule properties and disassembly [73, 159]. In budding yeast, the essential Cdc48 protein controls SG removal [159]. The loss of Ubx2p or Vms1p, Cdc48/VCP cofactors, impairs SG clearance [159].

VCP also controls granule clearance in mammalian cells. To this end, VCP associates with SGs, which is dependent on the stressor, cell type, and PTMs. SUMOylation in the N-domain increases upon oxidative or ER stress. Concomitant with SUMOylation, VCP relocates to SGs and the nucleus [140]. Notably, the stress-induced redistribution is abolished for several pathogenic VCP mutations (G97E, R155C, R159H, A232E; Table 4). Some of these mutants (G97E, R155C, R159H) also diminish the assembly of active VCP hexamers and alter cofactor binding [140, 153, 220, 221].

In C2C12 myoblasts, the overexpression of *VCP*_*R155H*_ or *VCP*_*A232E*_, mutant genes linked to multisystem proteinopathies (MSP, see below) triggers SG formation even without stress. These abnormal granules contain TDP-43, a key component of neuronal inclusions. VCP_R155H_ and VCP_A232E_ compromise the disassembly of arsenite-SGs in C2C12 cells [67]. By contrast, the mutants do not prevent the removal of heat shock-SGs. At the same time, pharmacological VCP inhibitors interfere with the dissolution of heat shock-SGs in HeLa cells [219].

Ubiquitination is indispensable for cells to recover from heat stress [68]. This requirement is shared by several cell types, including neurons. In particular, the VCP-dependent disassembly of heat shock-SGs relies on the ubiquitination of SG proteins. This prerequisite is not observed for arsenite-SGs.

Links between VCP and pathologic inclusions were uncovered by analyzing a superoxide dismutase 1 (SOD1) variant that causes ALS. The E3 ligase Dorfin (double ring finger protein, RNF19A) ubiquitinates SOD1_G85R_, but not wild type SOD1 [76]. Dorfin binds VCP directly, and VCP-Dorfin complexes are formed in vitro and in vivo*.* Following Dorfin-dependent ubiquitination, SOD1_G85R_ is degraded. Dorfin-mediated SOD1_G85R_ ubiquitination requires VCP; VCP_K524A_ (ATPase activity reduced compared with wild type VCP) interferes with this step [76]. However, VCP_K524A_ does not prevent the interaction between Dorfin and SOD1_G85R_. Importantly, Dorfin and VCP co-localize in neuronal inclusions of postmortem brain tissue obtained from PD and ALS patients [76]. This study supports the idea that during neurodegeneration VCP regulates the accumulation of inclusions by directly controlling the ubiquitination of misfolded proteins (Table 4).

**Table 4:** Characteristics of pathogenic VCP variants relevant to neurodegeneration. The properties of VCP mutants linked to neurodegeneration are listed. Phenotype descriptions focus on VCP activity, subcellular distribution, and the cellular activities impacted. The changes relative to the wild type protein are listed. Neurodegenerative diseases linked to the VCP mutants are also shown. Not determined, consistent results describing the effects on cellular parameters are not yet available; ?, disease link not defined. ALS, amyotrophic lateral sclerosis; CMT2Y, Charcot-Marie-Tooth disease, type 2y; HSP, hereditary spastic paraplegia; MSP1, multisystem proteinopathy 1; PMA, progressive muscular atrophy, a subtype of ALS. Information was accumulated from ClinVar or other databases [222–224] and original papers. *VCP* mutagenesis identified additional amino acid residues that modulate VCP function [225]. Only mutations linked to neurodegenerative diseases are included in Table 4

VCP variant	Phenotype: changes in cellular parameters	Disease link	References
**Mutations in N domain (residues 1–187)**			
I27V	not determined	MSP1, PD	[226, 227]
K60R	not determined	ALS	[228]
R89Q	not determined	ALS	[229]
N91Y	not determined	ALS- PMA, FTD, ALS, MSP1	[224, 229]
R93C	not determined	MSP1, ALS	[224, 228–230]
R93H	not determined	HSP, FTD, ALS, MSP1	[224, 231]
R95C	not determined	?	[223]
R95G	accelerated substrate unfolding in complex with UFD1L-NPL4; elevated basal ATPase activity; smaller increase in ATPase activity upon substrate binding; imbalanced cofactor binding; reduced nuclear levels; altered ER organization; impaired dendritic spine formation; reduced interaction with Ankrd13A; reduced interaction with caveolin-1	MSP1	[168, 220, 224, 232, 233]
R95H	not determined	?	[223]
G97E	suppresses VCP hexamer assembly	MSP1, CMTY2, FTD, ALS, atypical MSP1	[140, 224, 234]
D98E	not determined	MSP1	[223, 224]
D98V	not determined	ALS	[229]
I114V	not determined	ALS	[229, 235]
T127A	not determined	FTD	[234]
G128A	not determined	likely pathogenic	[223, 224]
P137L	accumulation of autophagosomes	MSP1	[236]
P137S	not determined	AD	[223, 224]
I151V	not determined	ALS	[229]
R155C	reduced nuclear levels; increased death of spinal cord motor neurons; aberrant synapse formation; altered transcription; ER stress; mitochondrial swelling; reduced mitochondrial membrane potential; reduced ATP production; reduced mitochondrial ATP synthase activity; reduced ADP/ATP translocation across mitochondrial membranes; increased oxidative stress; TDP-43 mislocalized; increased levels of insoluble and phosphorylated TDP-43 in brain; autophagosome-lysosome dysfunction (accumulation of autophagosomes and endolysosomes)	ALS, HSP, ALS- PMA	[174, 224, 228, 229, 237–240]
R155G	not determined	?	[223]
R155H	increased affinity for UFD1L-NPL4; accelerated substrate unfolding in complex with UFD1L-NPL4; elevated basal ATPase activity; smaller increase in ATPase activity upon substrate binding; reduced binding to UBXD1 (UBXN6); imbalanced cofactor binding; reduced nuclear levels; reduced mitochondrial membrane potential; reduced ATP production; excessive degradation of mitofusin; impaired mitochondrial fusion; altered axonal transport of mitochondria (*Drosophila* ortholog mutant dVCP R152H); deficient lysosomal clearance; reduced interaction with ANKRD13A; reduced interaction with caveolin-1	MSP1, ALS	[64, 136, 153, 173, 174, 220, 224, 232, 233, 241–243]
R155P	reduced nuclear levels	MSP1, FTD, ALS	[136, 224]
R155S	not determined	MSP1, FTD, A:S	[224]
G156C	not determined	ALS	[229]
G157R	accumulation of autophagosomes	MSP1, FTD, ALS	[224, 236]
M158V	increased number of spinal motor neurons with VCP-positive nuclei; increased levels of cytoplasmic TDP-43	ALS, MSP1, FTD	[224, 244]
R159C	VCP- and ubiquitin-positive cytoplasmic and nuclear aggregates in muscle	MSP1 HSP, ALS	[224, 229, 245–247]
R159G	not determined	ALS, ALS-FTD	[224, 229, 241]
R159H	accelerated substrate unfolding in complex with UFD1L-NPL4; elevated basal ATPase activity; smaller increase in ATPase activity upon substrate binding; increased cytoplasmic abundance of TDP-43	MSP1, ALS, FTD	[224, 229, 232, 244, 248]
R159S	not determined	ALS	[224]
S171R	not determined	CMT2Y	[249]
E185K	defective autophagy, accumulation of immature autophagosomes	CMT2Y	[250]
**Mutations in N-D1 linker (residues 188–207)**			
R191G	not determined	ALS	[229]
R191Q	accelerated substrate unfolding in complex with UFD1L-NPL4; elevated basal ATPase activity; smaller increase in ATPase activity upon substrate binding; reduced nuclear levels; increased cell death; aberrant synapse formation, altered transcription; TDP-43 mislocalized; ER stress; mitochondrial swelling; reduced mitochondrial membrane potential; reduced ATP production; reduced mitochondrial ATP synthase activity; reduced ADP/ATP translocation across mitochondrial membranes; increased oxidative stress	MSP1, ALS, PD, CMT2Y, FTD	[174, 224, 232, 237, 240, 251]
R191P	not determined	ALS, FTD	[224, 229]
L198W	accelerated substrate unfolding in complex with UFD1L-NPL4; elevated basal ATPase activity; imbalanced cofactor binding; smaller increase in ATPase activity upon substrate binding; cytoplasmic and intranuclear inclusions in muscle; deficient lysosomal clearance	MSP1	[65, 153, 232, 252]
**Mutations in D1 domain (residues 208–458)**			
I216M	not determined	?	[223]
A232E	increased affinity for UFD1L-NPL4; accelerated substrate unfolding in complex with UFD1L-NPL4; elevated basal ATPase activity; imbalanced cofactor binding; smaller increase in ATPase activity upon substrate binding; reduced nuclear levels; excessive degradation of mitofusin; impaired mitochondrial fusion; altered axonal transport of mitochondria (*Drosophila* ortholog mutant dVCP A229E); ubiquitin- and TDP-43-positive aggregates accumulated in muscle; TDP-43 accumulated in cytoplasm of brain cells; deficient lysosomal clearance; NF-kB activation; reduced interaction with ANKRD13A; reduced interaction with caveolin-1; altered processing of transcription factor SREBP1, changes in lipid biosynthesis	MSP1	[43, 65, 119, 136, 153, 173, 224, 232, 233, 242, 252]
T262A	increased affinity for UFD1L-NPL4; accelerated substrate unfolding in complex with UFD1L-NPL4; elevated basal ATPase activity; smaller increase in ATPase activity upon substrate binding	MSP1, PD	[232, 253]
N387H	TDP-43 positive inclusions in muscle	MSP1	[253]
N387T	not determined	ALS	[229, 247]
G376E	not determined; likely pathogenic	FTD	[254]
D395A	reduced ATPase activity	behavioral FTD	[255]
D395G	reduced ATPase activity; accumulation of tau-containing NFTs; increased spread of proteopathic seeds	FTD	[119, 224, 256–258]
A439S	not determined	MSP1	[259]
D450V	not determined	MSP1	[232]
**Mutations in D2 domain (residues 481–761)**			
R487H	not determined	ALS, pyramidal ALS	[229, 260]
E578Q	substrate trapping; ATPase activity deficient; ER stress induced; ubiquitinated proteins accumulated at ER membrane; deficient lysosomal clearance	MSP1	[64, 119, 173, 261]
D592N	impaired binding to 20S proteasome subunit	ALS, FTD with neurofibrillary tangles	[224, 229, 241, 262]
R662C	not determined	ALS	[229, 247]
**Others**			
Splice variant; c.1696-3C > T	not determined	ALS	[228]

VCP cofactors can direct the ATPase to SGs and thereby regulate granule removal [74, 75]. For example, the zinc finger AN1-type containing 1 (ZFAND1) colocalizes with arsenite-SGs, but not heat shock-SGs [75]. VCP needs ZFAND1 to associate with arsenite-SGs, but this is not the case for heat shock-SGs. Furthermore, *ZFAND1* knockdown delays the clearance of arsenite-SGs, but not of heat shock-SGs. The VCP_R155H_ mutant does not aggravate the effect of ZFAND1 depletion in HeLa cells, suggesting that ZFAND1 and VCP are part of the same pathway mediating SG clearance [75]. ZFAND1 also recruits the proteasome, which likely collaborates with VCP to dissolve arsenite-SGs [75].

Genetic and pharmacological studies implicate the autophagy activating kinase 1/2 (ULK1/2) in the removal of heat shock-SGs via VCP phosphorylation and activation [141]. ULK1/2 and VCP colocalize in heat shock-SGs. Heat stimulates their interaction and increases the ULK1/2-mediated phosphorylation of VCP on S13, S282, and T761 [141]. ULK1/2 inhibition slows down SG dissolution, but has no effect on SG assembly. Moreover, phospho-mimetic, but not phospho-defective, VCP mutants restore SG disassembly when *VCP* or *ULK1* are knocked down [141]. Finally, the ULK1/2 agonist LYN1604 accelerates SG removal. LYN1604 also facilitates the clearance of persistent SGs that contain pathogenic mutant proteins, such as VCP_A232E_, FUS_R521C_, or TIA1_A381T_ [141]. In addition, the small molecule SMER28 activates VCP and boosts the removal of pathological variants of huntingtin or ataxin-3, and of cellular inclusions that contain ubiquitinated proteins [107]. SMER28 achieves the clearance of aberrant proteins by stimulating autophagosome biogenesis and UPS function [107].

VCP not only regulates SG removal, it also controls granule formation. Thus, VCP knockdown impairs SG assembly in HeLa cells exposed to arsenite, heat shock, or the proteasome inhibitor MG132 [73]. Similar results were obtained (i) with pharmacological VCP inhibitors (Eeyarestatin I, ML240), and (ii) the knockdown of VCP cofactors UFD1L (ubiquitin fusion degradation 1 like) or PLAA (phospholipase A2-activating protein) [73]. VCP, UDF1L, and PLAA also determine the composition of SGs. Their depletion prompts the accumulation of defective ribosomal products (DRIPs) adjacent to or in SGs. Conversely, DRIPs are absent from control granules. In addition, VCP regulates the size and distribution of SGs [73].

Aggregates of α-synuclein, tau, or TDP-43 can serve as templates to trigger aggregate formation in the cytoplasm of neighboring cells. VCP reduces such proteopathic seeding in neurons and thereby limits the spread of pathological inclusions in the CNS. To achieve this, VCP likely detects seed-induced lysosomal damage and stimulates the removal of dysfunctional lysosomes [256]. VCP mutations, for example VCP_D395G_, can interfere with the elimination of aberrant lysosomes. As a result, the dissemination of proteopathic seeds is enhanced. Furthermore, the diminished ATPase activity of VCP_D395G_ compromises its disaggregase activity. VCP_D395G_ has been linked to behavioral FTD and neuronal tau aggregates that resemble AD neurofibrillary tangles [119, 257, 258]. The partial dissolution of tau aggregates present in human AD-brains is fueled by ATP and relies on tau poly-ubiquitination [119].

Despite solid evidence linking *VCP* mutations to neurodegenerative diseases, it should be emphasized that their pathologies and the properties of intracellular aggregates vary widely. Information on the presence of VCP in pathologic inclusions can be conflicting, even for the same disease. Such discrepancies may arise from differences in the disease stage, cell types examined, genetic, or environmental variables. On the other hand, wild type and mutant VCP variants can differ in their association with pathological inclusions [119].

To date, several general statements summarize the contributions of VCP to granulostasis. (a) The specific role of VCP is dependent on the cell type. (b) VCP modifications, especially SUMOylation and phosphorylation, regulate granulostasis. (c) The type of acute stress determines how VCP affects granule dynamics. (d) Pathogenic VCP variants can alter the dynamics, clearance, or formation of granular compartments. (e) Wild type VCP restricts the spread of proteopathic aggregates. (f) VCP’s role for granules/aggregates formed during chronic stress is poorly defined.

#### Effects of VCP mutations on glial cells

In the context of neurodegeneration, much attention has been given to the function of VCP in neurons. Glial cells ensure the survival and proper functioning of neurons [263]. Recent studies examined VCP in glial cells [237, 264, 265]. The impact of mutant VCP may vary in neurons and astrocytes, although mutations (R155C, R191Q) affect both cell types [237]. In astrocytes, mutant VCP can have cell-autonomous as well as non-cell-autonomous effects. For instance, mutant VCP may prompt the cell-autonomous reactive transformation of astrocytes [265]. The inability to support wild type motor neurons in a co-culture system illustrates the non-cell-autonomous effects of VCP mutant astrocytes [237]. It will be interesting to determine the impact of VCP mutant astrocytes in a spinal cord environment.

#### VCP and lipid metabolism

The formation of SGs and lipid droplets is closely intertwined, and the stress-induced SG assembly is commonly accompanied by the production of LDs [266]. Interestingly, VCP also regulates the accumulation of fat and LDs in cultured liver cells and experimental mice [43]. In particular, VCP_A232E_ alters lipid homeostasis in cultured hepatocytes and mice on a high fat diet [43]. The VCP-dependent mechanisms that determine the proteolytic processing and nuclear transport of the transcription factor SREBP1 are discussed in a previous section.

The AMFR-INSIG1-VCP complex controls the sterol-dependent degradation of HMG-CoA reductase, which is a rate-limiting enzyme for the biosynthesis of cholesterol [267, 268]. In a cell culture model, the ATPase negative double mutant VCP_K251Q/K524Q_ is unable to support the sterol-dependent degradation of HMG-CoA reductase [267].

In addition, ceramides worsen the pathologies linked to VCP_R155H_ [269]. However, feeding pregnant mice with a diet enriched in lipids ameliorates the lethal effects caused by homozygous *VCP*^*R155H/R155H*^ in the offspring [270].

### VCP and neurodegenerative diseases

**Fig. 3:**
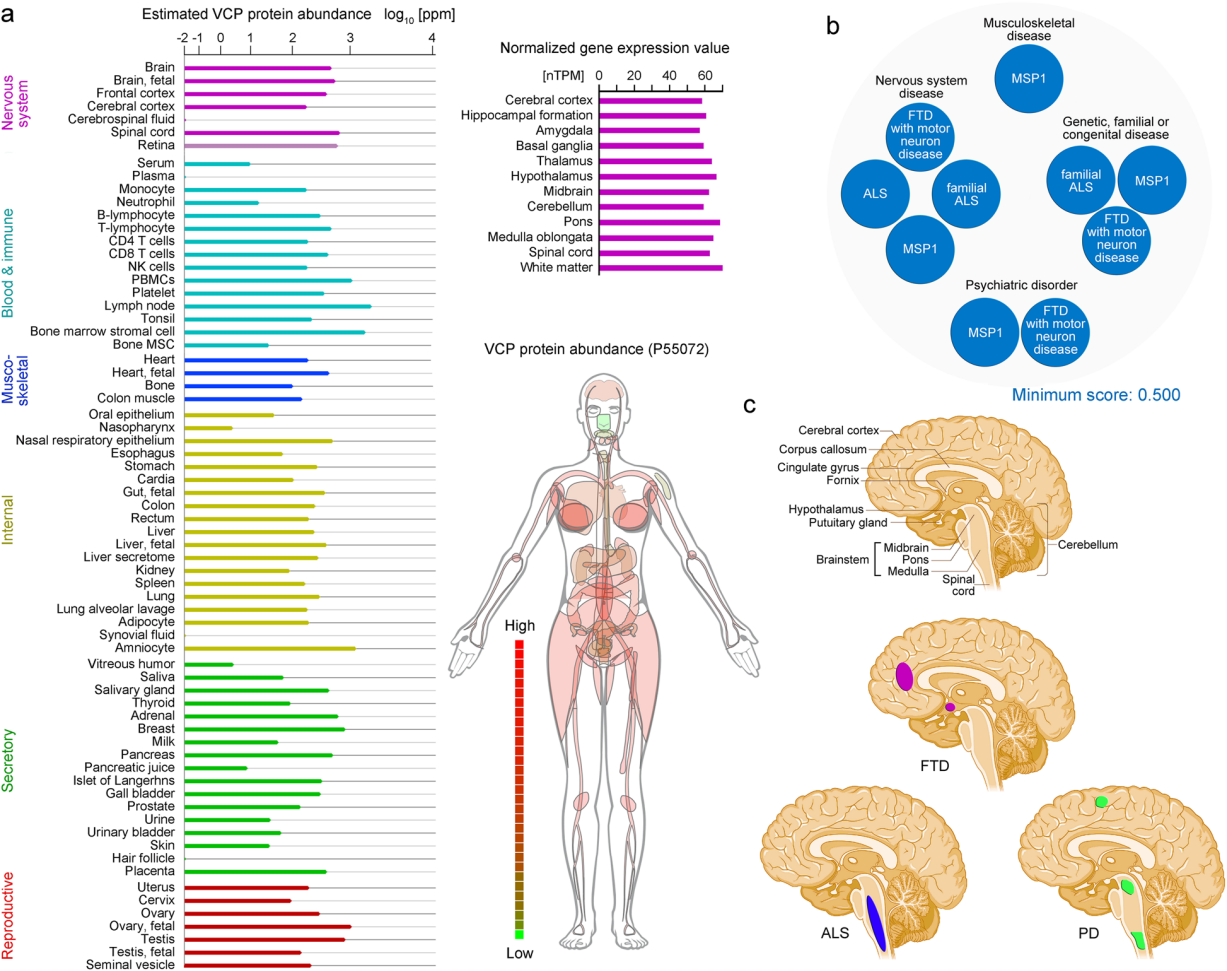
VCP protein in different tissues, *VCP* transcripts in the nervous system, and VCP-associated diseases. **a** The abundance of the VCP protein is shown for different tissues and cell types (MSC, mesenchymal stromal cell; NK cells, natural killer cells; PBMC, peripheral blood mononuclear cell). The *VCP* gene expression in different parts of the brain and the distribution of the VCP protein throughout the human body are depicted [24, 271, 272]. **b** VCP disease associations with a minimum score of 0.500 are presented. They belong to different categories [273]. **c** Brain regions that are particularly affected by FTD, ALS, or PD are delineated in color. Not all of the regions altered by the disease are demarcated

VCP is implicated in a broad spectrum of health conditions that include several neurodegenerative diseases. Information pertinent to transcript and especially protein concentration in the healthy and diseased nervous system are important for strategies aimed at disease prevention and treatment. The VCP protein levels in various tissues are depicted in Fig. 3a. More details are provided in Table S3.

#### *VCP* transcripts and protein in nervous system

The *VCP* gene is expressed ubiquitously, but the transcript and protein levels vary somewhat according to the tissue and cell type. This includes *VCP* transcripts in different brain regions [271, 274]. *VCP* transcripts undergo alternative splicing [97], but the physiological relevance of splice variants and their possible protein products are poorly understood. Among 36 human *VCP* transcripts, 15 encode proteins that comprise between 55 and 806 amino acid residues [275]*.*

The VCP protein is present throughout the body, with high concentrations in most organs and tissues (Fig. 3a). In the nervous system, VCP protein abundance is particularly low in the cerebrospinal fluid (Fig. 3a). By contrast, the VCP protein is abundant in glial and neuronal cells of the cerebral cortex, Purkinje cells of the cerebellum, and hippocampal neurons (Fig. 3, Table S3 [271]).

#### VCP mutations associated with neurodegenerative disease

The *VCP* gene is located on chromosome 9; many of the clinical variants are inherited in an autosomal-dominant fashion. Several not mutually exclusive scenarios are possible in the context of neurodegeneration; (i) mutant *VCP* causes disease, and/or (ii) mutant *VCP* modifies disease onset and progression. Pathological VCP variants can trigger inclusion body myopathy associated with Paget disease of the bone and frontotemporal dementia (IBMPFD); this rare disease is also described by the more general term multisystem proteinopathy (MSP) [276]. VCP_R155H_ is the most common variant linked to MSP1, whereas VCP_A232E_ causes especially serious pathologies [173]. *VCP* mutations can instigate other neurodegenerative conditions [277], such as amyotrophic lateral sclerosis (ALS [278]), Parkinson’s disease (PD [253, 279]), Charcot-Marie-Tooth disease type 2y (CMT2Y [249, 250, 253]), and hereditary spastic paraplegia (HSP [280]). Patients with *VCP* mutations display a wide spectrum of phenotypes. In a previous study, ~ 9% of the patients presented with clinical manifestations of ALS, 4% with PD, and 2% with AD [281]. However, as illustrated by ALS [229], VCP mutants make different contributions to neurodegeneration among global populations. Aside from causing neurodegeneration, *VCP* variants also modulate the pathologies of Alzheimer’s disease (AD) and some polyglutamine (polyQ) diseases, such as Huntington’s disease (HD) and spinocerebellar ataxia type 3 (SCA3/Machado-Joseph disease) [28, 119, 121].

The diseases linked to *VCP* mutations affect distinct brain regions (Fig. 3). Nevertheless, these conditions share the accumulation of intracellular or extracellular inclusions that contain ubiquitinated proteins. The buildup of such aggregates is consistent with impaired protein quality control and disturbed proteostasis [282]. It is poorly understood how VCP mutations lead to the variety of phenotypes and defects in different parts of the brain. Diagnosis and treatment are further complicated by marked interfamilial and intrafamilial variations of symptoms [281].

Table 5 summarizes key features of the neurodegenerative diseases that can be caused by *VCP* mutations.

**Table 5:** Neurodegenerative conditions that can be caused by *VCP* mutations. Clinical attributes were collected from GeneReviews® [283]; they may overlap for some of the VCP diseases. For most neurodegenerative diseases listed in the table, different VCP variants can serve as trigger. (See Table 4 for details on the link between individual VCP mutants and the diagnosed type of neurodegeneration.) Not determined, consistent datasets are not available

Disease; prevalence	Proportion attributed to pathogenic variants of *VCP* gene	Clinical features	Affected parts of the nervous system; functions impaired
MSP1 (IBMPFD); prevalence approximately 1 per 300,000 [283],	> 99%	early-onset Paget disease of the bone; adult-onset proximal and distal muscle weakness; premature frontotemporal dementia; cause of death includes respiratory and cardiac failure[276, 284]	degeneration of the frontal and anterior temporal lobes; control of reasoning, personality, movement, speech, social behavior, language; marginal impact on episodic memory
ALS; prevalence approximately ~ 2–9 per 100,000 [285]	1–2% of familial ALS [241] (5–10% of ALS cases are familial)	upper and lower motor neuron dysfunction; rapid and progressive paralysis; disease presentation, progression, and survival highly variable; death mostly due to respiratory failure	affects brain and spinal cord; motor neurons and potentially additional areas in frontal and temporal lobes; systems outside the nervous system (bone, muscle); cognitive and/or behavioral symptoms possible
PD; global prevalence 200 per 100,000 [286]	~ 4% of MSP1 cases with features of PD [276, 281]	postural instability, tremor, rigidity, bradykinesia [286]; falls and pneumonia as major causes of death	loss of dopaminergic neurons in the substantia nigra, motor dysfunction
CMT2Y; prevalence < 1 per 1,000,000 [287]	rare [276, 288]	peripheral neuropathy; distal muscle weakness and atrophy; distal sensory loss [289]	axonal neuropathy, non-demyelinating
Hereditary spastic paraplegia, SPG8; global prevalence 1 per 1,000,000 [287]	not determined	progressive lower-limb spasticity; SPG8 generally more severe than other forms of HSP [290]	not determined
Behavioral FTD	not determined	early-onset progressive cognitive impairment; apathy; irritability; progressive speech–language impairment, culminating in mutism; bulimia; epileptic seizures	not determined
Vacuolar tauopathy (VCP_D395G_) [119]	not determined	frontotemporal lobar degeneration accompanied by tau inclusions (FTLD-tau); no muscle or bone disease comorbidity [119]	tau aggregates in degenerating frontal neocortex; vacuolization in non-degenerating brain regions (i.e. visual cortex)

#### Multisystem proteinopathy 1 (MSP1)

VCP mutations can cause the rare disorder inclusion body myopathy (IBM) associated with Paget’s disease of the bone (PBD) and frontotemporal dementia (IBMPFD). Formerly called inclusion body myopathy associated with Paget’s disease of the bone and frontotemporal dementia (IBMPFD), the disease is now referred to as multisystem proteinopathy 1 (MSP1) [291, 292]. Almost all cases (> 99%) are caused by mutations in the *VCP* gene. MSP1 is characterized by three pathological features, early-onset Paget disease of the bone, adult-onset proximal and distal muscle weakness, and premature frontotemporal dementia (FTD; Table 5, [283]). However, 2 to 3% of the patients who carry pathogenic *VCP* mutations show only frontotemporal dementia [257]. Aside from the *VCP* mutation, environmental factors may also determine the MSP1 pathology [277]. This hypothesis is supported by established links between patient environments and the severity of Paget’s disease of the bone [293], ALS [278], and PD [294].

At the cellular level, the disease is characterized by ubiquitin-positive inclusions containing RNA-binding proteins, such as TDP-43, in the CNS, bones, and muscles [292]. *VCP* mutations related to MSP1 are located in the N-domain, the linker between the N-domain and D1-domain, and the D1-domain (Fig. 2, [295]). Several of these mutations increase the VCP ATPase activity of the D2 domain, but compromise autophagy. Impaired autophagy leads to the accumulation of autophagosomes and autophagic markers in the inclusions [163, 292, 295]. The dysregulation of autophagy may result from the abnormal binding of mutant VCP to substrates and cofactors [295].

Multiple MSP1 mutants display impaired VCP nuclear localization and SG association [136, 140]. Specifically, mutations R95G, R155H, R155P, R155C, R191Q, and A232E reduce the nuclear abundance of VCP [136]. VCP controls mitochondrial homeostasis; and several mutants exhibit mitochondrial dysfunction (Table 4). The pathologies of MSP1 patients align with the essential role of VCP to support organ and tissue functions. Both brain and muscle heavily rely on proper mitochondrial performance.

#### Amyotrophic lateral sclerosis (ALS)

Amyotrophic lateral sclerosis (ALS) is a multisystem neurodegenerative condition, and 5–25% of all patients develop advanced FTD [296]. Approximately 9% of MSP1 patients show clinical manifestations of ALS [276]. ALS is characterized by the progressive loss of motor neurons in the brain or spinal cord. Other motor and non-motor domains also contribute to the disease [263, 297, 298]. Most ALS cases are sporadic (sALS, 90–95%), whereas 5 to 10% are familial (fALS). Among familial cases, 1 to 2% are linked to autosomal-dominant *VCP* mutations [237, 241]. While amounting to less than 1% of sALS cases, *VCP* mutations are also found in sALS [247, 299].

At the cellular level, more than 95% of ALS patients show TDP-43 redistribution to the cytoplasm and aggregate formation. This includes ALS-related *VCP* mutations, which commonly interfere with the nuclear localization of TDP-43 [237, 239, 300]. Interestingly, the ATP-competitive VCP inhibitor ML240 can reverse the mislocalization of TDP-43 in VCP-mutant motor neurons [301]. On the other hand, overexpression of *Ter94*, the *Drosophila* ortholog of *VCP*, rescues motor neuron degeneration instigated by the knockdown of *TBPH* (encodes *Drosophila* TDP-43) [302].

VCP mutation may also redistribute fused in sarcoma (FUS) from the nucleus to the cytoplasm in motor neurons derived from induced pluripotent stem cells (iPSCs) [303]. The knockdown of *Cabeza* (*Caz*), the *Drosophila* ortholog of human *FUS*, induces ALS-related pathologies, including motor neuron degeneration [304]. The defects are exaggerated by *ter94* loss-of-function mutations. By contrast, wild type *ter94* overexpression rescues the phenotypes [304].

Together, the experiments in flies emphasize that the RNA-binding proteins TDP-43 and FUS as well as VCP collaborate to maintain neuronal homeostasis.

Astrocytes are key players in the progression of neurodegenerative diseases. In ALS, *VCP* mutant astrocytes undergo reactive transformation in a cell-autonomous fashion [237]. The state differs for *VCP* and *SOD1* mutant astrocytes, emphasizing that the causative mutation determines the contribution of glial cells to ALS pathology [265].

#### Parkinson’s Disease (PD)

Parkinson’s Disease (PD) is characterized by the formation of amyloid inclusions, known as Lewy bodies. These inclusions arise from misfolded α-synuclein and contain VCP [305]. Lewy body formation is accompanied by the loss of dopaminergic neurons in the substantia nigra and motor dysfunction [263].

Worldwide, the prevalence of PD amounts to 200 cases per 100,000 individuals [286]. Genetic predisposition combined with environmental factors is the most common cause of PD; 5 to 10% of the cases represent monogenic forms of the disease [306]. With ~ 4% incidence, PD is an established attribute of IBMPFD [281]. Unilateral rigidity, tremor, and bradykinesia are clinical features of PD patients with *VCP* mutations [251, 286]. Mutant VCP can initiate FTD and motor neuron disease. However, these cases are rare; they account for less than 1% of the mutations linked to FTD and movement disorders [279].

The cellular pathophysiology of PD includes impaired protein clearance, mitochondrial defects, and neuroinflammation [307], processes related to VCP dysfunction [308]. Notably, *VCP* gene expression declines at preclinical and early clinical stages of PD [308].

#### Hereditary Spastic Paraplegia (HSP)

Hereditary Spastic Paraplegia (HSP) describes a group of neurological disorders that are distinguished by the degeneration of upper motor neurons [309]. While motor neuron degeneration is part of the HSP and ALS pathology, several parameters of the diseases differ. In general, the disease onset is earlier for HSP (mean age: 30–40 years) when compared with ALS (mean age: 65 years) [310]. Weakness in the lower limbs is commonly symmetric for HSP patients, but asymmetric for ALS [310]. HSP patients display genetic and clinical heterogeneity, but are commonly linked to mutations that affect endolysosomal and autophagic processes [311]. Through its interaction with strumpellin (SPG8, KIAA0196, WASHC5), VCP is connected to Autosomal Dominant Spastic Paraplegia Type 8 [97]. The *VCP* mutations R155C and R159C have been identified in patients diagnosed with spastic paraplegia [238, 245].

### Charcot-Marie-Tooth disease, type 2y (CMT2Y)

Aside from the CNS, VCP also controls the performance of the peripheral nervous system. This is illustrated by Charcot-Marie-Tooth (CMT) disease, a hereditary neuropathy marked by chronic motor and sensory polyneuropathy [289]. *VCP* mutations are linked to axonal CMT disease (CMT2Y) [249, 250], and the dominant mutant VCP_E185K_ compromises autophagy. One outcome of the mutation is the buildup of immature autophagosomes [250]. How VCP_S171R_, another mutant causing CMT2, impacts cellular homeostasis has yet to be defined [249].

### Modifiers of VCP disease

The clinical manifestations differ widely for MSP1 patients. Modifier genes, such as *APOE* variants may contribute to this heterogeneity [312]. Other potential modifiers of MSP1 have been reviewed recently [277]. Some of these candidate modifiers are linked to neurodegenerative diseases. This is exemplified by the RNA-binding protein TIA1 [313]. Under stress conditions, TIA1 nucleates the assembly of SGs [214]. Mutations in the low-complexity domain of TIA1 alter the dynamics of SGs [313]. The TIA1-containing granules interact with TDP-43, which becomes insoluble. As TDP-43 associates with VCP as well as TIA1 [12], it is conceivable that *TIA1* mutations aggravate the proteostasis defects caused by *VCP* variants.

Genetic variants of VCP cofactors may also function as modifiers of VCP disease. For example, optineurin (OPTN) is part of a neuroprotective network that involves neurons, microglia, and oligodendrocytes [314]. OPTN binds ubiquitinated substrates, controls vesicle trafficking, inflammatory signaling, autophagy, mitophagy, and necroptosis [314–316]. Proximity labeling identified VCP and several VCP cofactors as OPTN binding partners [317]. Interestingly, several *OPTN* mutations are associated with sALS and fALS [314].

To date, solid scientific evidence supports the conclusion that different components of the VCP network, when mutated, cause neurodegeneration. However, the limited number of MSP1 patients, combined with the complexity of the VCP network, and the heterogeneity of patient populations, complicate the identification of genetic modifiers of VCP disease. While several VCP mutants function as drivers of human disease, they may also modify the severity of neurodegenerative disorders (see below).

### VCP variants as modulators of neurodegenerative disorders

*VCP* classifies as a disease susceptibility gene, and *VCP* variants may function as genetic modifiers. This concept is illustrated by disease-causing mutations that affect the nervous system. For example, VCP modulates the pathological phenotypes associated with *ATXN3* mutations, which can cause spinocerebellar ataxia type 3 (Machado-Joseph Disease) [318, 319]. *VCP* is also a candidate modifier gene for neurofibromatosis type 1 [320], ALS-related motor neuron degeneration [302, 304], and hereditary spastic paraplegia caused by atlastin mutations [167].

Furthermore, *VCP* functions as a modifier of poly(GR) translation in *Drosophila* [321]. Poly(GR) dipeptide repeats can be produced when the *C9ORF72* gene carries G_4_C_2_ expansions. Poly(GR) peptides are cytotoxic and may cause ALS/FTD. RQC mechanisms (see above) that are mediated by VCP limit the accumulation of poly(GR) peptides. In particular, RQC relies on VCP phosphorylation by the kinase Akt (reviewed in [175]), which phosphorylates residues Ser352, Ser746, and Ser748 [322]. Notably, mutations of VCP Ser352, Ser746, or Ser748 are linked to neurodegeneration (Fig. 2).

In line with the scenarios described above, we speculate that *VCP* mutants also modulate the manifestations of primary disease-causing events. For instance, the severity of FTD disease elicited by mutant VCP cofactors, such as SVIP [66], is modified by *VCP* gene variants. Mutations of PTM sites, such as residues phosphorylated by Akt [322], may also impinge on the course of neurodegeneration. Finally, VCP variants may have a broad impact on human health and functions of the nervous system by changing the age of onset and other disease phenotypes [323, 324].

#### Alzheimer’s disease (AD)

AD has a global prevalence of ~ 51.6 per 1,000,000 [325]. The disease is characterized by amyloid-β inclusions and neurofibrillary tangles, which contain hyperphosphorylated tau [326]. Tau stabilizes microtubules and is degraded by the proteasome or autophagy [327, 328]. The accumulation of hyperphosphorylated tau in neurons promotes neuronal degeneration, loss of synapses, and cognitive deficits [329]. In the frontal cortex of AD patients, VCP is part of a MAPK/metabolism network; the ATPase colocalizes both with neurofibrillary tangles and Aβ plaques [330]. AD plaques also contain ubiquitin [331], a binding partner of various VCP complexes.

To our knowledge, there is no solid evidence for the idea that *VCP* mutations are a primary cause of AD. However, VCP contributes to the AD phenotype. This is illustrated by the interplay between VCP and tau. First, the drop of VCP concentration in the AD cortex is accompanied by a rise in tau phosphorylation [332]. Second, *VCP* knockdown in primary rat cortical neurons increases the abundance of Ser262/356-phosphorylated tau, while reducing soluble tau [332]. Third, the mutant VCP_D395G_ is linked to autosomal-dominant dementia characterized by neuronal tau aggregation [119]. As the ATPase activity of VCP_D395G_ is impaired, the mutant fails to disaggregate tau inclusions properly.

#### PolyQ expansion diseases

PolyQ diseases are caused by the expansion of cytosine-adenine-guanine (CAG) repeats in specific genes [333]. This results in extended polyQ tracts in pathogenic proteins and induces the formation of toxic polyQ aggregates [333].

Huntington’s disease (HD) and spinocerebellar ataxia type 3 (SCA3, also called Machado-Joseph disease) are caused by extensive polyglutamine (polyQ) expansion of the huntingtin or ataxin-3 protein [334]. The *VCP* gene modifies the severity of both diseases. VCP directly binds expanded polyQ proteins, including mutant huntingtin, ataxin-1, ataxin-7, and androgen receptor [335]. Cells that produce aberrant polyQ proteins concentrate VCP in the nucleus. However, as the interaction of VCP with DNA repair proteins is compromised, the abundance of DNA double-strand breaks increases [335].

##### Huntington’s disease

With a prevalence of ~ 1 per 10,000 in Western countries [336], Huntington’s disease (HD) is characterized by the progressive neurodegeneration of the caudate nucleus and putamen, parts of the basal ganglia [297]. Patients suffer from motor disturbances and cognitive decline. In rare cases, patients carry a combination of mutant VCP and mutant forms of huntingtin (mtHtt [337]).

The mtHtt protein can have a higher affinity for VCP than its wild-type counterpart [121]. It recruits VCP to mitochondria, where the ATPase triggers mitophagy and ultimately cell death [121]. VCP can also compromise mitochondrial function by excessive degradation of myeloid cell leukemia sequence 1 (MCL1), a mitochondrial outer membrane protein [52]. VCP knockdown and gossypol, a nonspecific VCP inhibitor, have beneficial effects in HD models [52, 338]. As well, the overexpression of *NPL4* and *UFD1* ameliorates the polyQ protein toxicity in yeast and mammalian cells [23]. In neurons that synthesize pathogenic polyQ proteins, such as mtHtt or ataxin-3, VCP carries a specific pattern of PTMs (phospho-S612, phospho-T613, acetyl-K614). The modifications promote aberrant VCP nuclear localization, histone H3 and H4 deacetylation, and compromise the VCP-dependent transcriptional control [339].

##### Spinocerebellar ataxia type 3

The global prevalence of SCA3, a hereditary neurodegenerative disorder, amounts to 1–5 in 100,000 [340]. It is caused by an extensive polyQ expansion of the protein ataxin-3 [341, 342]. Almost all SCA3 patients with cerebellar ataxia have difficulty speaking, eye conditions, and vestibular malfunction. Motor neuron degeneration is frequent and may affect upper and lower motor neurons [342].

Ataxin-3 functions as a deubiquitinase (DUB) that associates with VCP. Ataxin-3 and UFD1 compete for the association with VCP [28]. Thus, ataxin-3 binding reduces the interaction of VCP with ubiquitinated clients and the retrotranslocation of ERAD substrates. Pathological polyQ expansion of ataxin-3 enhances VCP binding and culminates in the impairment of ERAD [28]. In addition, the VCP/ataxin-3 complex participates in the DNA damage response by removal of the E3 ubiquitin ligase RNF8 [343] and in the regulation of early stages of autophagy [164].

### VCP disruptors

Another group of variants that are linked to neurodegeneration and VCP are represented by mutations that indirectly alter VCP function. Here, we refer to them as VCP disruptors. For instance, G_4_C_2_ expansions in the *C9ORF72* gene result in cytotoxic dipeptide repeat products, such as poly(GA) proteins. Poly(GA) aggregation sequesters VCP and can culminate in the onset of ALS or FTD [344, 345].

An alternative route to VCP disruption is the generation of new binding proteins. This scenario is illustrated by a mutation in *ATP7A*, which encodes a copper-transporting ATPase [346]. The *ATP7A* mutation uncovers a UBX domain, supports a novel VCP-ATP7A interaction, and leads to adult-onset isolated distal motor neuropathy.

### VCP as actionable target for neurodegenerative diseases?

#### Biomarkers

Aside from genetic testing, no dependable biomarkers are available to diagnose MSP1 [288, 347]. *VCP* mutations commonly cause a relocation of TDP-43 to cytoplasm for MSP1. However, this is not limited to MSP1, but also observed for other neurodegenerative diseases.

#### Ethnic background, sex-specific differences

The prevalence of VCP diseases and clinical manifestations may vary according to the ethnic background and sex of an individual [234, 348]. For instance, the prevalence of PDB is low in Asian countries when compared to Western populations [349]. Recent data also indicate sex-specific differences, at least in the context of a specific ethnic background and *VCP* variant [348].

Publicly accessible information on the clinical manifestations and other parameters of VCP disease ranges from case reports to large scale and systematic evaluation of patient data. The size of patient cohorts differs widely among these analyses. Individual studies assess VCP disease in multiple countries [350], or focus on Asia [229, 234, 351–364], Europe [255, 365–370], Australia [371], and Hispanic [348] or African American [372] patients. Table S4 summarizes key results for several publications.

To date, the published work suggests that the contributions of *VCP* mutations to neurodegenerative disease depend on the characteristics of the patient cohort. Thus, the genetic or ethnic background and the geographical location of patients can impact the trajectory of VCP disease. This is illustrated by *VCP* mutation frequencies as risk factors for ALS [278]. They were determined as 0.8% in European populations, but only as 0.3% in Asia [278]. Sex-specific differences are also emerging for some VCP variants [348]. The molecular mechanisms through which biological and genetic traits, ethnicity, or sex impact VCP disease remain largely undefined.

#### Pharmacological compounds, dietary intervention

Drugs and other pharmacological agents can alter the ATPase activity of VCP [107, 373]. This includes compounds that stimulate the ATPase activity of the D1 domain [107]. Small molecule inhibitors that bind VCP directly may interfere with diverse VCP activities and have side effects [95, 347]. Adverse effects may be tolerable if systemic VCP inhibition occurs over a limited period of time, for example to boost the elimination of cancer cells. By contrast, VCP-associated neurodegeneration relies on long-term treatment to delay onset or mitigate disease progression. This scenario requires alternative treatment regimens. Small molecule protein–protein-interaction modulators are particularly promising, as they can target a selected fraction of the VCP protein interaction network [374]. Here, drug development could be guided by VCP complexes whose function is disease-relevant and altered by a specific *VCP* mutation. Adaptor-specific antibody fragment inhibitors are alternatives to small molecule inhibitors. The approach is feasible, as demonstrated with antibody fragments that interfere with VCP-p47 complex formation [375]. Small molecules or antibody fragments that target individual VCP cofactors may also be useful to enhance a set of VCP-dependent functions.

The use of pharmacological agents could be strengthened by personalized diet plans. Given the links of VCP to lipid metabolism, nutritional interventions tailored to the patient’s *VCP* mutation may delay the onset and pathogenesis of VCP disease.

#### Clinical trials and clinical studies

In May 2023, six clinical trials listed on the NIH Clinical Trials website were related to VCP (or p97) and neurodegeneration (see Table S5; [376]). Three of these trials were associated with ALS or pre-symptomatic ALS, one with MSP1 (listed as IBMPFD), one focused on behavioral FTD, and one was a patient registry for rare diseases. Five of the studies were classified as “observational”. The “interventional” study on ALS patients (NCT03367650) included a “dietary supplement”. For none of the listed trials are results available on ClinicalTrials.gov.

Furthermore, the VCP inhibitors CB-5083 and CB-5339 are part of clinical trials for the treatment of different malignancies. The two trials assessing CB-5083 have been terminated; one of two trials evaluating CB-5339 has been withdrawn. A second trial on CB-5083 (Phase 1) is listed as recruiting participants (Table S5). As of May 2023, no additional trials related to both MSP1 and VCP were published by the EU, Australian, or WHO International Clinical Trials Registries [377–379].

Examples of other clinical trials potentially relevant to MSP1 are summarized below. A trial on patients with acute central retinal artery occlusion (JPRN-UMIN000023979) suggests that compounds targeting VCP could address specific aspects of organ dysfunctions, especially related to the eye [380, 381]. In-depth analyses are needed to determine whether *VCP* variants that cause MSP1 also have VCP-dependent effects on ocular health.

A proof-of-concept trial was conducted with patients suffering from sporadic inclusion body myositis [382]. Arimoclomol induces the heat shock response and had promising effects in mice overexpressing the human *VCP* mutant (A232E). A clinical trial evaluated the adverse outcomes in human subjects over 12 months (NCT00769860). It included 24 participants age > 50 years; 16 were treated with arimoclomol (2/16 participants withdrew) and 8 received a placebo. Details on the *VCP* variants in the patient population were not provided. Overall, the trial did not reveal significant benefits for inclusion body myositis when patients treated with arimoclomol [383].

Aside from registered trials, clinical studies also shed light on the diverse pathological manifestations of VCP disease. The individuals with clinical manifestations of VCP disease were examined for a cohort of 32 carriers of mutant *VCP*. In this patient group, 43.5% displayed cardiovascular complications [384]. The patients developed cardiovascular dysfunctions at later stages of VCP disease [384].

A link between VCP abundance and skin disease emerged recently [385]. For a group of 25 patients with psoriasis, epidermal VCP levels gradually raise from control to psoriatic skin regions [385]. The epidermis, dermis, and adnexa of the skin are characterized by elevated VCP abundance. Notably, proximal muscle weakness and psoriasis may manifest in the same patient [386]. To our knowledge, it has not been explored to which extend VCP disease variants play a role in skin disease.

So far, clinical trials offer only limited support for the hypothesis that targeting VCP variant proteins alone will benefit patients with VCP disease.

### Care for patients with VCP disease

An in-depth discussion of patient care is beyond the scope of our review. Details and links to more comprehensive publications are provided in the section below.

VCP-related MSP1 is a rare disease. It often affects multiple organ and cellular systems and is heterogeneous with respect to disease onset and symptoms [387]. To date, there is no unifying concept that describes the role of *VCP* mutations in neurodegenerative diseases. The VCP Standards of Care Working Group has developed guidelines for the diagnosis, treatment, and clinical surveillance of patients with VCP-associated disease [288, 347]. Given the complexity of the clinical manifestations, genetic testing remains the most reliable method to identify *VCP* mutations as the underlying cause of disease. In recent years, marked progress has been made for all aspects of MSP1 patient care. Giving more weight to the ethnic background, sex, environmental factors, and nutritional interventions [387] could further improve the quality of care.

### Future directions

#### Knowledge gaps

Despite extensive research on the role of VCP in neurodegeneration, considerable knowledge gaps remain to be addressed. The development of better theranostic strategies requires the concerted effort in multiple disciplines. Textbox 1 lists some of the outstanding questions.

**Table:** Outstanding questions

***VCP biology and disease***
• Which VCP-dependent functions are directly linked to the pathologies observed for VCP-induced neurodegeneration?
• How does the “VCP code” of PTMs affect disease onset and progression? Is VCP-sumoylation relevant to the disassembly of toxic aggregates?
• Can the nucleocytoplasmic distribution be modulated for VCP mutants that fail to enter the nucleus?
• How similar are VCP-induced inclusion bodies in muscles to aggregates in the nervous system? How similar are these inclusions to stress granules?
• Are inclusions generally detrimental to cell survival, or do they help to sequester toxic protein aggregates?
• Inclusion bodies in the muscles of MSP1 patients are an early sign of disease. Do the protein aggregates spread to the nervous system, either through secretion or *via *exosomes?
• What is the role of ciliary VCP for brain health?
• Which VCP activities are modulated by sex?
***Disease phenotypes***
• Why does MSP1 preferentially affect muscle, bone, and the nervous system?
• What determines the chronological sequence of clinical manifestations in different organs and tissues?
• How does the composition of VCP networks determine the impact of VCP variants on different cell types, tissues, and organs? Is the MSP1 phenotype determined by the availability of VCP cofactors or binding proteins?
• Are distinct parts of the nervous system especially vulnerable to the combination of a *VCP* mutation with specific variants of modifier gene(s)?
• Which VCP interacting proteins determine the onset, progression, or pathology of a specific *VCP* mutation?
***Role of non-neuronal cells in MSP1-mediated neurodegeneration***
• How do VCP mutations alter the biology of glial cells in the CNS and PNS?
• How do glial cells with *VCP* mutations contribute to the pathologies of MSP1?
• What are the non-autonomous effects of *VCP* mutations outside of neurons and astrocytes?
***Therapeutic interventions***
• Do VCP PTMs provide druggable targets? Is the targeting of VCP regulators, such as ULK1/2, a suitable approach for a subset of VCP mutants?
• Is the targeting of astrocytes and other glial cells a mandatory step to prevent neurodegeneration in MSP1 patients?
• What are the different parameters that contribute to the heterogeneity of MSP1 clinical manifestations? Which are relevant to patient care?
• Can a personalized “scoresheet” of the VCP variant, genetic modifiers, sex, and environmental factors instruct on optimal patient care? Can the “scoresheet” be used to prevent or delay disease onset or progression?
• What dietary and other non-drug interventions can improve the health of MSP1 patients?

#### Future studies

**Fig. 4:**
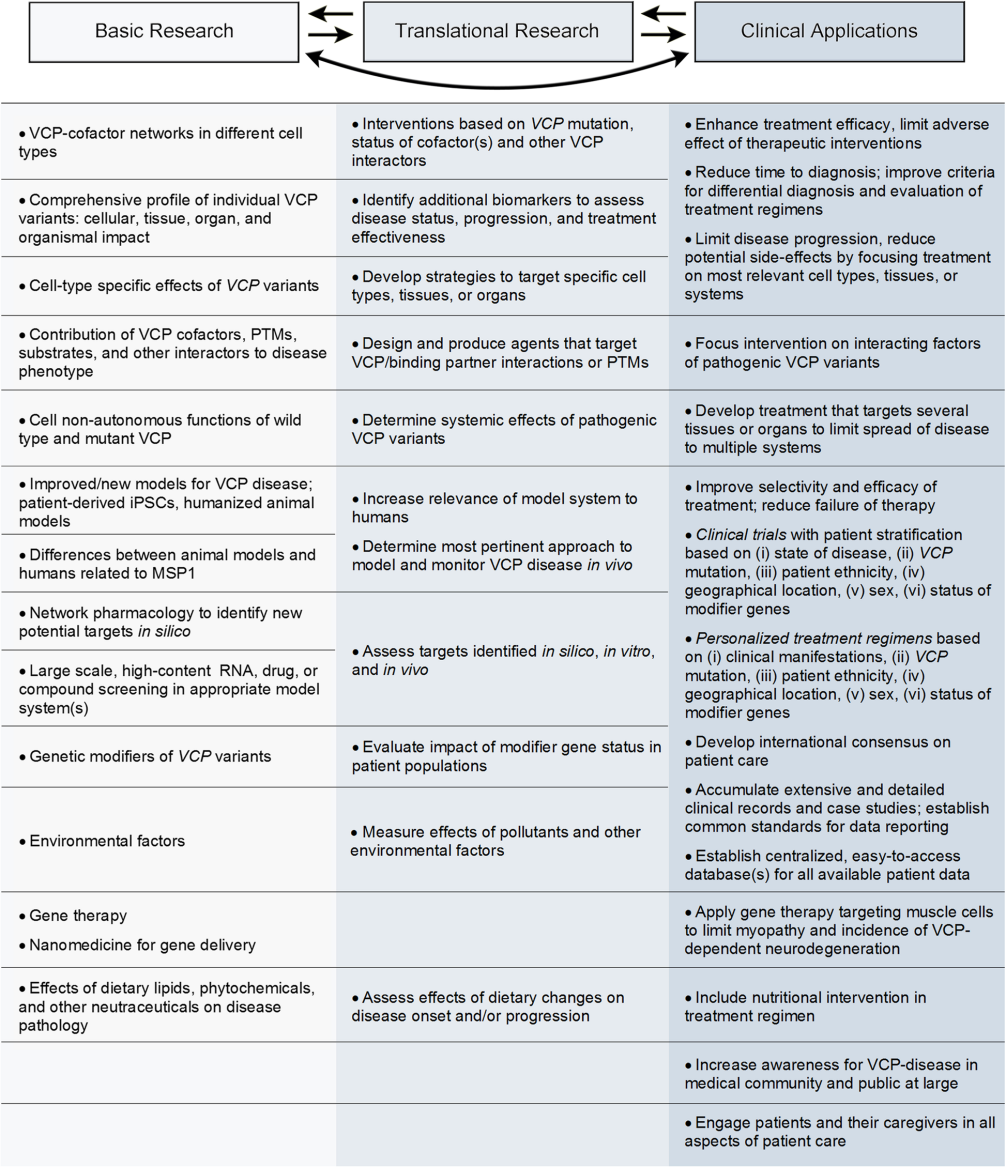
Future directions to advance knowledge and theranostics in the field of VCP disease. The figure highlights the integrative approach that is driven by continuous feedback among different disciplines. Key issues that have to be addressed in the near future are depicted. They are related to basic research, translational research, and clinical applications. The list is not comprehensive and has to be updated on a regular basis

Based on the open questions (Textbox 1), we speculate on the trajectory the field of VCP disease will take in the short-term. To achieve the ultimate goal, better patient care, the VCP community has to attend to diverse topics (Fig. 4). In our opinion, several areas of investigation are critical to propel the field forward. They include -but are not limited to- a better understanding of VCP disease heterogeneity, non-cell autonomous effects of VCP variants, and identifying new candidate targets for therapeutic intervention. Moreover, as phenotypes of VCP mutants may vary in humans and experimental animals [388], disease models have to be improved and expanded. Other topics in need of attention relate to the ethnic and geographical differences of patient populations, genetic modifiers, and environmental factors. Given that MSP1 is a rare disease, answering these questions poses a challenge.

The recommendations for VCP patient care are continuously updated [288]. At the same time, it is necessary to boost awareness about MSP1 among medical professionals, patients, and their families. This is particularly urgent in communities where medical facilities are not available or difficult to access.

Finally, the complexity of MSP1 and the current knowledge gaps offer ample opportunity for innovation. For instance, the combination of gene therapy and nanomedicine could advance the treatment of MSP1. In particular, gene silencing, gene transfer, or genome editing in muscle cells may limit the severity of myopathy. These approaches are promising for Duchenne muscular dystrophy [389] and ALS [390]. They can be further improved with inert nanocarriers that circumvent the adverse effects of viral vectors [391, 392]. Thus, nano-based gene therapy could become a pioneering clinical application to control the pathology in the muscles and other tissues of MSP1 patients.

## Conclusions

The links between *VCP* mutations and neurodegenerative diseases are well-established. While inclusion bodies are a hallmark of the VCP pathology, their formation, composition, and dynamics are far from understood. Aggregates associated with neurodegeneration generally develop under conditions of chronic oxidative stress. Most cell and animal models do not adequately mimic these conditions. Future studies to improve the models and their relevance to human disease are needed. Ideally, these models incorporate patient-derived cells and multiple cell types, such as neurons and different glial cells.

Much effort has been put into the development of compounds that bind VCP and inhibit or activate its ATPase activity. However, the ubiquitous expression of the *VCP* gene and the numerous biological activities that require VCP argue against this strategy in the context of neurodegeneration. A more focused approach, illustrated by the targeting of VCP-cofactor complexes, may have fewer side effects and better outcomes when long-term treatment is necessary.

Taken together, we anticipate that a multipronged approach will generate novel insights into the molecular mechanisms underlying MSP1. Major advancements require collaborations that include basic researchers, clinicians, patients, and their caregivers. The effort of interdisciplinary and multinational teams will be mandatory to translate new knowledge into better care for MSP1 patients.

### Supplementary Information

**Additional file 1: Figure S1.** Alignment of VCP posttranslational modifications and VCP variants. **Table S1.** VCP complexes and their contribution to biological processes. **Table S2.** Information on key VCP binding proteins. **Table S3.** Human tissues with high VCP protein abundance. **Table S4.** Identification or evaluation of VCP patients from different geographical locations.

**Additional file 2: Supplemental Table 5.** Clinical trials relevant to VCP disease.

## Data Availability

Not applicable.

## Abbreviations

AD: Alzheimer’s disease
ALS: Amyotrophic lateral sclerosis
ANKZF1: Ankyrin repeat and zinc finger peptidyl tRNA hydrolase 1, VMS1 in budding yeast
ATL1: Atlastin 1, GTPase; Sey1 in budding yeast
C9ORF72: Chromosome 9 open reading frame 72
CCT: Chaperonin containing tailless complex polypeptide 1
CHIP: Carboxyl terminus of Hsp70-interacting protein
CMT2Y: Charcot-Marie-Tooth disease, type 2y
CNS: Central nervous system
DRIP: Defective ribosomal product
DRP: Dipeptide repeat proteins
ELDR: Endo-lysosomal damage response
ER: Endoplasmic reticulum
ERAD: Endoplasmic reticulum-associated degradation
FUS: Fused in sarcoma
HD: Huntington’s disease
HSP: Hereditary spastic paraplegia
IBMPFD: Inclusion body myopathy associated with Paget disease of the bone and frontotemporal dementia
iPSC: Induced pluripotent stem cell
LD: Lipid droplet
MAD: Mitochondria-associated degradation
Mito TAD: Mitochondrial protein translocation associated degradation
MSP: Multisystem proteinopathy
NFT: Neurofibrillary tangles
OPTN: Optineurin
PAAL: Phospholipase A2-activating protein, Doa1
PD: Parkinson’s disease
PMA: Progressive muscular atrophy
PNS: Peripheral nervous system
QC: Quality control
RQC: Ribosome-associated protein quality control
SOD1: Superoxide dismutase 1
SREBP1: sterol regulatory element binding factor 1
TER94: Transitional endoplasmic reticulum ATPase 94
TDP-43: TAR DNA-binding protein 43
TRiC: Tailless complex polypeptide 1 ring complex
UPS: Ubiquitin-proteasome system
VCP: Valosin containing protein, p97, Transitional Endoplasmic Reticulum ATPase 94 (TER94), CDC48
