# Supplementary material for: Valosin containing protein (VCP): initiator, modifier, and potential drug target for neurodegenerative diseases
Source: Mol Neurodegener. 2023 Aug 7;18:52. doi: 10.1186/s13024-023-00639-y (PMC10405438; doi:10.1186/s13024-023-00639-y)
Supplement: Supplementary file 1 — Additional file 1: Figure S1. Alignment of VCP posttranslational modifications and VCP variants. Table S1. VCP complexes and their contribution to biological processes. Table S2. Information on key VCP binding proteins. Table S3. Human tissues with high VCP protein abundance. Table S4. Identification or evaluation of VCP patients from different geographical locations. [file 13024_2023_639_MOESM1_ESM.pdf]

Supplemental Information

Valosin containing protein (VCP): initiator, modifier, and potential drug target for neurodegenerative diseases

Siwei Chu[1], Xinyi Xie[1], Carla Payan[1] and Ursula Stochaj[1, 2]

Department of Physiology[1] & Quantitative Life Sciences Program[2], McGill University, Montreal, Canada

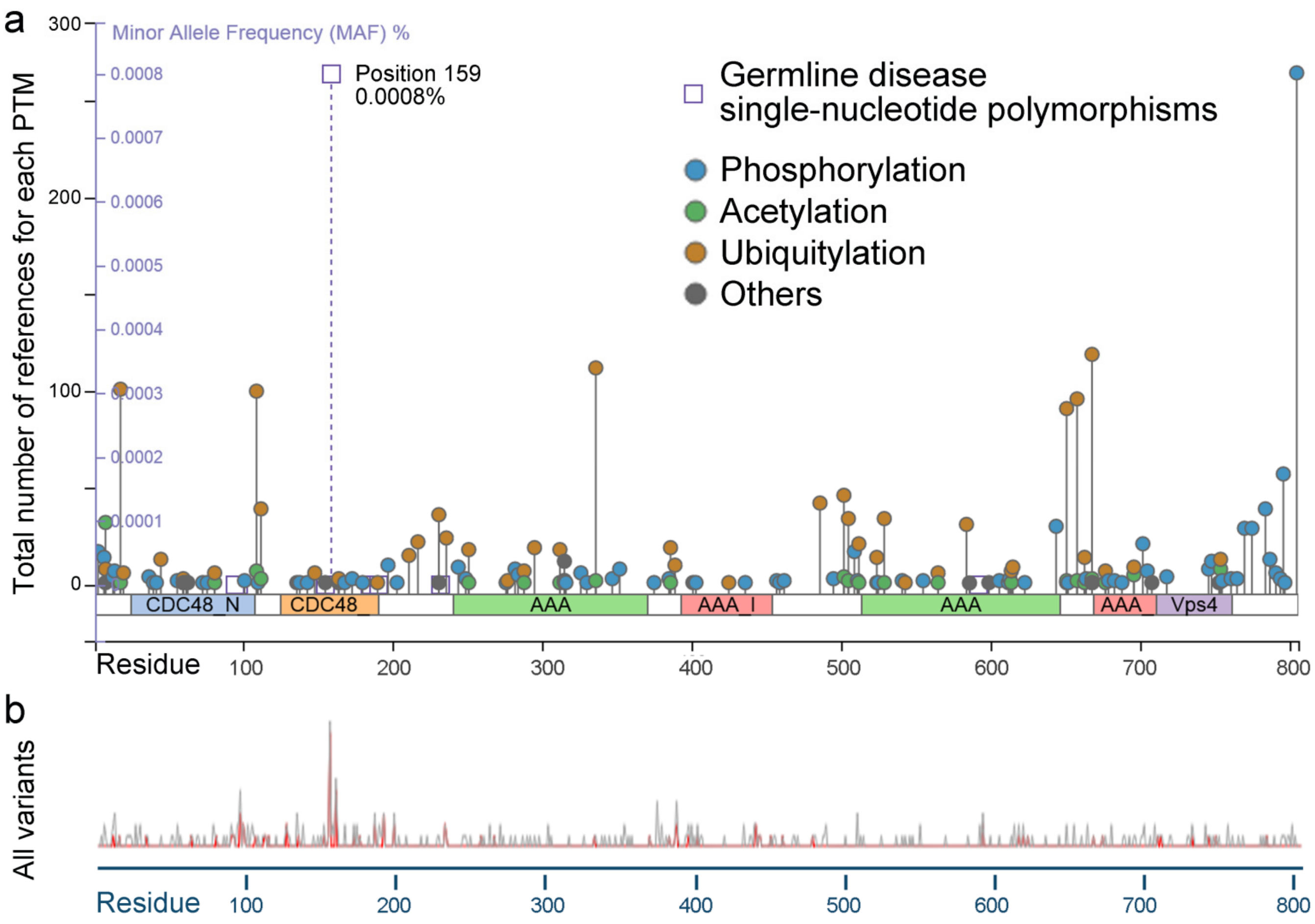

Figure S1. Alignment of VCP posttranslational modifications and VCP variants. (a) PTMs listed for human VCP on PhosphoSite Plus [1] are shown. The number of references is indicated for individual PTMs (x-axis). Protein residues linked to germline single-nucleotide polymorphisms (SNPs) are indicated as blue squares. Position 159 has the highest minor allele frequency (MAF). Other germline disease SNPs have been identified for position 95, 97, 185, 191, 232, and 592. The minor allele frequencies for these positions are currently unknown. Functional domains of VCP are depicted at the bottom of the graph [2]. Details on the domains are available through the Pfam database. (b) The distribution of all VCP variants is aligned with the location of PTMs. See Fig. 2 of the main text for additional information.

Table S1. VCP complexes and their contribution to biological processes. The information on VCP complexes in mammalian cells was collected from different sources [3-5].

Complex	Biological functions of complex	Organism
VCP homohexamer	proteasomal degradation (UPS), retrograde transport; vesicle fusion; unfolded protein response (ER quality control)	Mouse
SNARE complex (STX5, VCP, NSFL1c)	protein binding; protein transport; ER to Golgi transport; vesicle fusion; synaptic vesicle exocytosis; ER; Golgi	Rat
VCIP135-NSFL1C/p47-VCP	promotes Golgi membrane fusion	Human
VCIP135-STX5A-NSFL1C/p47-VCP	promotes Golgi membrane fusion	Human
VCP-UFD1-NPL4-IP3 receptor	proteasomal degradation (UPS); protein binding; ER	Rat, mouse
VCP-UFD1-NPL4	proteasomal degradation (UPS); unfolded protein response (e.g. ER quality control); cytoplasm	Rat
p47-VCP	vesicle fusion; Golgi; cytoplasm	Rat
p47-VCP	protein targeting, sorting and translocation; protein transport; vesicle fusion	Rat
BRCA1-VCP	DNA repair; nucleus	Human
Profilin 1 complex (cytoplasmic actin 2, clathrin heavy chain 1, hsc70, profilin 1,VCP, tubulin-β2)	assembly of protein complexes; endocytosis; actin cytoskeleton	Human, mouse
SVIP-VCP-DERL1	unfolded protein response (ER quality control); ER	Human
AMFR-VCP-DERL1	proteasomal degradation (UPS); unfolded protein response (ER quality control); ER	Human
DERL1-VCP-VIMP	ERAD	Human
VCP-VIMP-DERL2	unfolded protein response (ER quality control); ER	Human
VCP-VIMP-DERL1-DERL2-HRD1-SEL1L	unfolded protein response (ER quality control); ER	Human
VCP-UFD1-NPL4	proteasomal degradation (UPS); unfolded protein response (ER quality control); ER	Rat
VCP-VIMP-DERL1-DERL2-HRD1-SEL1L	unfolded protein response, ERAD	Human
Membrane protein complex (VCP, UFD1L, SEC61B)	not listed in Harmonizome database	Human
Membrane protein complex (DERL1, NPLOC4, UFD1L, VCP, VIMP)	ERAD	Human
SELK multiprotein complex (RPN1, RPN2, CANX, DDOST, STT3A, VCP, SELENOS, DERL1, DERL2, SELENOK)	unfolded protein response, ERAD	Human
Ubiquilin-VCP-erasin	ERAD, response to ER stress	Human
AMFR-VCP-NGLY1	ERAD	Mouse

AMFR-VCP-UBXN1	ERAD	Mouse
NGLY1-VCP-UBXN1	ERAD	Mouse
AMFR-VCP-UBXN1-NGLY1-RAD23B	ERAD	Mouse
VCP-SELK-DERL1	unfolded protein response (ER quality control); ERAD	Human
VCP-VIMP-DERL2	ER	Human
GP78-INSIG1-VCP	membrane; negative regulation of cholesterol biosynthesis	Hamster

AMFR, autocrine motility factor receptor, E3 ubiquitin protein ligase, GP78, RNF45;
INSIG, insulin induced gene 1, CL-6;
NGLY1, N-glycanase 1, CDG1V, PNG1, PNGase;
SELS/VIMP, selenoprotein S, VCP-interacting protein;
SELK, selenoprotein K;
Additional alternative names are listed in the main text.

Table S2. Information on key VCP binding proteins. Proteins interacting with human VCP have been sorted according to their organ and tissue distribution. Note that some VCP binding proteins have high abundance in several locations. The table lists the name of the VCP binding protein, the *gene*, and the aliases of the VCP binding partner. Cellular pathways associated with the VCP interacting protein, the abundance of the interacting protein and of its transcripts are also shown. The tissue distribution of cofactors was assembled with The Human Protein Atlas [6] for top VCP interactors as identified with BioGrid [7]. Information on pathways and transcripts was curated from The Human Protein Atlas, The GeneCards Suite, and UniProt [6, 8, 9]. DUB, deubiquitinating enzyme; PBMCs, peripheral blood mononuclear cells; UPS ubiquitin-proteasome system.

VCP binding partner; protein, gene, alias	Pathway	Tissues with abundant VCP interacting proteins	Tissues with abundant transcripts for VCP interacting proteins
**VCP interacting protein detected in brain, highly abundant in cerebellum**			
NPL4, *NPLOC4*	UPS, ERAD	cerebellum, nasopharynx, bronchus, liver, gall bladder, urinary bladder, testis, appendix	skeletal muscle
DNA-dependent metalloprotease Spartan, *SPRTN*	DNA repair	cerebellum	testis, skeletal muscle, bone marrow, thymus
UBXD2, erasin, *UBXN4*	ERAD	multiple tissues, including cerebellum (Purkinje cells), caudate nucleus (neurons)	liver, skeletal muscle, thymus, parathyroid gland, thyroid gland
Ubiquitin thioesterase OTU1, *YOD1*, *DUBA8*, *OTUD2*	UPS, ERAD	multiple tissues, including cerebellum (cells in granular layer, Purkinje cells)	bone marrow, esophagus
Ankyrin repeat and zinc finger domain-containing protein 1, *ANKZF1*, *ZNF744*	mitochondrial stress response, likely ERAD	cerebellum (Purkinje cells), testis (cells in seminiferous ducts), Fallopian tube (glandular cells), smooth muscle (smooth muscle cells)	cerebellum, cerebral cortex, lymph node, pituitary gland, seminal vesicle
VCP lysine methyltransferase, *VCPKMT*	trimethylates VCP on K315, reduces VCP ATPase activity	medium abundance for most tissues, including cerebellum (cells in granular layer, Purkinje cells)	immune system (B cells)

**VCP interacting protein detected in brain, highly or medium abundant in cerebral cortex or other brain regions**			
Ubiquitin C, *UBC*	UPS, protein degradation, DNA repair, cell cycle control, endocytosis, cell signaling	high in multiple tissues, including cerebral cortex (glia), cerebellum (cells in granular and molecular layer), caudate nucleus (glia)	skeletal cells
AN1-type zinc finger protein 2B, *ZFAND2B*	UPS	high levels in multiple tissues, including brain; cerebral cortex (glia, neurons), cerebellum (cells in molecular layer, Purkinje cells), hippocampus, (glia, neurons), caudate nucleus (neurons)	granulocytes, small intestine, T-cells, NK cells, duodenum
E3 ubiquitin-protein ligase synoviolin, *SYVN1, HRD1, DER1*	UPS, ERAD	cerebral cortex, cerebellum, hippocampus, lung, salivary gland, stomach, duodenum, gall bladder, pancreas, testis, Fallopian tube, cervix, placenta, lymph node, tonsil	liver, pancreas, salivary gland, tonsil
Ubiquilin-1, *UBQLN1*	UPS, autophagy, ERAD	high levels in multiple tissues, including brain; cerebral cortex (neurons), cerebellum (cells in molecular layer, Purkinje cells), caudate nucleus (neurons)	skeletal muscle, thymus
COP9 signalosome subunit 5, *COPS5*	UPS, controls ubiquitin conjugation	high levels in most tissues, including brain, cerebral cortex (endothelial cells, glia, neurons), cerebellum (cells in granular and molecular layers, Purkinje cells), hippocampus (glia), caudate nucleus (glia)	skeletal muscle, heart muscle, cerebral cortex, spinal cord, dendritic cells
Phospho-lipase A-2-activating protein, *PLAA*	UPS, ubiq-uitin-medi-ated mem-brane protein trafficking	cerebral cortex (neurons), testis (preleptotene spermatocytes, spermatogonia), bone marrow (hematopoietic cells)	skeletal muscle, bone marrow, thymus, heart muscle
Derlin-1, *DERL1, DER1*	UPS, ERAD	cerebral cortex (neurons), adrenal gland, nasopharynx, GI tract (stomach, duodenum, small intestine, colon), liver, pancreas, testis, seminal vesicle, Fallopian tube, placenta, appendix, lymph node, tonsil	liver, skeletal muscle, T-cells, bone marrow, lymph node
Huntingtin, *HTT*	possibly microtubule-mediated transport, vesicle function	cerebral cortex	cerebral cortex, thyroid gland, pancreas, skin, cerebellum

small VCP interacting protein, *SVIP*	UPS, ERAD	high in multiple tissues, including cerebral cortex (neuropil)	spinal cord, pons and medulla, midbrain, thyroid gland, pituitary gland
UBX domain-containing protein 10, *UBXN10, UBXD3*	ciliogenesis	cerebral cortex (neurons), adrenal gland (glandular cells), nasopharynx (respiratory epithelial cells), bronchus (respiratory epithelial cells), Fallopian tube (glandular cells), placenta (trophoblastic cells)	Fallopian tube, testis
E3 ubiquitin-protein ligase *PRKN, PARK2*	autophagy, mitophagy, UPS	cerebral cortex (neurons), caudate nucleus (neurons), gall bladder (glandular cells), kidney (tubular cells), testis (Leydig cells)	multiple tissues, including several brain regions
E3 ubiquitin-protein ligase RNF31, *RNF31*	NF-κB activation, control of inflamma-tion	medium protein levels for most tissues	skeletal muscle, thymus, skin, spleen, cerebral cortex
Tether con-taining UBX domain for GLUT4, *ASPL, UBXD9, UBXN9*	retains GLUT4 in intracellular vesicles in the absence of insulin	medium protein levels for most tissues	liver, skeletal muscle, salivary gland, placenta, cerebral cortex
N-glycanase 1, *NGLY1, PNG1*	UPS, degly-cosylates denatured N-linked gly-coproteins in cytoplasm	high protein levels for multiple tissues, medium or low in brain	skeletal muscle, testis, dendritic cells
Ataxin-3, *ATXN3, SCA3*	UPS, con-trols degra-dation of misfolded chaperone clients, pro-teostasis, transcription, cytoskeleton regulation, myogenesis	brain with medium or low protein levels; most other tissues with high protein levels	skin, smooth muscle, monocytes, cerebellum, endometrium
UBX domain-containing protein 6, *UBXN6, UBXD1,*	ERAD; en-dosome to lysosome transport, macroau-	low tissue specificity	skeletal muscle, adrenal gland, seminal vesicle, cerebral cortex, epididymis, pancreas

UBXDC2	tophagy		
**VCP interacting proteins, more abundant outside of the nervous system**			
Ubiquitin recognition factor in ER-associated degradation protein 1, *UFD1, UFD1L*	UPS, ERAD, spindle disassembly at end of mitosis	breast, smooth muscle, skeletal muscle	T-cells, granulocytes, skeletal muscle
NSFL1 cofactor p47, *NSFL1C, UBXN2C*	Golgi fragmentation and re-assembly during mitosis	esophagus, kidney, testis, prostate, vagina, cervix, placenta, skin	skeletal muscle, granulocytes, cerebral cortex, skin
E3 ubiquitin-protein ligase AMFR, *RNF45, AMFR, GP78*	UPS, ERAD	duodenum, small intestine, kidney	skeletal muscle, liver, placenta, testis, kidney
Fas-associated factor, *FAF1, UBXD12, UBXN3A*	UPS, DNA replication	salivary gland, testis	skeletal muscle, testis, thymus
Selenoprotein S, *SELENOS, SELS, VIMP*	UPS, ERAD	duodenum, small intestine, colon, rectum, pancreas, placenta, appendix, tonsil	dendritic cells, pancreas, liver, salivary gland
UBX domain-containing protein 2A, *UBXN2A, UBXD4*	ubiquitin binding, acetylcholine receptor binding	bronchus, gall bladder, testis	skeletal muscle, heart muscle
FAS-associated factor 2, *FAF2, UBXD8, UBXN3B*	UPS, ERAD, lipid droplet degradation	parathyroid gland, GI tract, pancreas, testis, appendix	liver, parathyroid gland, placenta, testis, pituitary gland, thymus, ovary, basal ganglia, cerebral cortex
UBX domain-containing protein 1, *UBXN1, SAKS1*	negative regulation of ERAD and UPS, immune responses	testis, placenta	skeletal muscle, T-cells, B-cells, total PBMCs
Ubiquitin conjugation factor E4 B,	UPS, may control myosin assembly	pancreas, placenta, tonsil	skeletal muscle, cerebellum, corpus callosum

UBE4B, UFD2	in striated muscles		
UBX domain-containing protein 7, *UBXN7*	UPS, ubiquitin-binding adapter	thyroid gland, nasopharynx, epididymis, placenta	cerebellum, bone marrow, thymus
UBX domain-containing protein 8, *UBXN8, UBXD6*	ERAD	testis, ovary	ovary, liver, epididymis
BCL2-associated athanogene 6, *BAG6*	UPS	testis	testis
Breast cancer type 1 susceptibility protein, *BRCA1*	UPS, DNA repair, E3 ubiquitin ligase	skin (epidermal cells), lymph node (germinal center)	testis, thyroid gland, bone marrow, tonsil lymph node
Derlin-2, *DERL2, DER2*	UPS, ERAD	pancreas (exocrine glandular cells), epididymis (glandular cells), placenta (syncytiotrophoblast cells)	total PBMCs, pancreas, T-cells, granulocytes, dendritic cells
Selenoprotein K, *SELK, SELENOK*	ERAD, immune cell regulation	salivary gland	granulocytes, pancreas
UBX domain-containing protein 11, *UBXN11, UBXD5*	organization of actin cytoskeleton	nasopharynx (ciliated cells: axoneme, rootlets, tip of cilia), duodenum (glandular cells), pancreas (exocrine glandular cells), Fallopian tube (ciliated cells: axoneme, tip of cilia)	Fallopian tube, monocytes, testis, basal ganglia, epididymis
Ankyrin repeat domain-containing protein 13A, *ANKRD13A*	UPS, control of protein localization to endosome, control of receptor internalization	thyroid gland (glandular cells), Fallopian tube (ciliated cells: tip of cilia), appendix (lymphoid tissue), lymph node (germinal center cells), tonsil (germinal center cells)	testis, placenta, tonsil, smooth muscle, lymph node
E3 ubiquitin-protein ligase RNF19A, Double ring-finger protein, (Dorfin 1), *RNF19A*	UPS, E3 ubiquitin-protein ligase, ubiquitinates pathogenic SOD1 variants	colon (glandular cells), testis (cells in seminiferous ducts), breast (myoepithelial cells), smooth muscle (smooth muscle cells), bone marrow (hematopoietic cells), medium in brain	bone marrow and lymphoid tissue, lymph node, testis, cervix, liver, hypothalamus, tonsil, gall bladder

**Proteins involved in signaling**			
RAC-alpha serine/ threonine-protein kinase, *AKT1, PKB*	signaling; *in vitro* VCP phosphorylation on S352, S746, S748	multiple tissues, including cerebral cortex (neurons), cerebellum (cells in granular and molecular layer), hippocampus (neurons)	multiple tissues, including several brain regions; high in immune system
Serine/ threonine-protein kinase PINK1, mitochondrial, *PINK1*	autophagy, mitochondrial quality control, mitophagy	parathyroid gland (glandular cells), adrenal gland (glandular cells), rectum (glandular cells), placenta (trophoblastic cells), breast (myoepithelial cells)	skeletal muscle
Serine/ threonine-protein kinase SIK2 (salt-inducible kinase 2), *SIK2*	ERAD, fatty acid oxidation, autophagy, immune responses, glucose metabolism, protein serine/ threonine kinase	most tissues with medium protein levels	pancreas, skeletal muscle, adipose tissue, pituitary gland
**Inconclusive data, or low abundance in brain**			
Deubiquitinating protein VCPIP1, *VCPIP1, VCIP135*	DNA repair, re-assembly of Golgi apparatus and ER after mitosis	inconclusive data	bone marrow
UBX domain-containing protein 2B, p37, *UBXN2B*	Golgi biogenesis, ER biogenesis, VCP adaptor	inconclusive data	granulocytes, cerebellum, hippocampus, cerebral cortex, liver
Rhomboid-related protein 4, *RHBDD1*	ERAD	high levels in multiple tissues, but *not in brain*	thymus
Insulin induced gene 1, *INSIG*	ERAD, controls sterol-induced ERAD- of HMG-CoA reductase	data not available	liver, monocytes

Table S3. Human tissues with high VCP protein abundance. Data were compiled from The Human Protein Atlas [6]. Cell types with high levels of VCP protein are in parentheses.

**System**	**Organ, tissue**
Brain	Cerebral cortex (glia, neurons), cerebellum (Purkinje cells), hippocampus (neurons)
Endocrine tissues	Parathyroid gland (glandular cells)
Respiratory system	Nasopharynx (respiratory epithelial cells), bronchus (respiratory epithelial cells), lung (alveolar cells, macrophages)
Proximal digestive tissues	Oral mucosa (squamous epithelial cells), esophagus (squamous epithelial cells)
Gastrointestinal tract	Stomach (glandular cells), colon (endothelial cells, glandular cells, peripheral nerve/ganglion), rectum (glandular cells)
Gall bladder	Gall bladder (glandular cells)
Urinary system	Kidney (cells in glomeruli, cells in tubules), urinary bladder (urothelial cells)
Male tissues	Testis (early spermatids), epididymis (glandular cells), seminal vesicle (glandular cells), prostate (glandular cells)
Female tissues	Fallopian tube (glandular cells), endometrium (glandular cells), uterine cervix (squamous epithelial cells), placenta (decidual cells, trophoblastic cells), breast (adipocytes, glandular cells)
Adipose tissues	Adipose tissues (adipocytes)
Soft tissues	Soft tissues (fibroblasts)
Skin	Skin (fibroblasts, keratinocytes, Langerhans cells, melanocytes)
Lymphoid tissues, bone marrow	Appendix (glandular cells, lymphoid tissue), lymph node (germinal center cells, non-germinal center cells), tonsil (germinal center cells, non-germinal center cells, squamous epithelial cells), bone marrow (hematopoietic cells)

It should be noted that the VCP transcript abundance may differ from the abundance of the VCP protein. Additional details on the *VCP* transcript abundance can be found in The Tissues and GeneCards databases [8, 10]. Alternatively spliced versions of the *VCP* transcript are depicted by the GeneCards database [8].

Table S4. Identification or evaluation of VCP patients from different geographical locations. Studies that assess different global populations for *VCP* mutations are listed. The analyses range from large-scale genome sequencing to case studies with a limited number of patients. The list is not meant to be comprehensive; it illustrates the diverse approaches used to monitor *VCP* variants in different global populations. ALS, amyotrophic lateral sclerosis; fALS, familial ALS; FTD; frontotemporal dementia; FTLD, frontotemporal lobar degeneration; IBM, inclusion body myopathy; PDB, Paget's disease of the bone; sALS, sporadic ALS.

**Origin or geographical location of probands**	Size of cohort	Female/ Male [%]	Main results relevant to VCP disease	Refer-ences
**International group** (USA, Ireland, Bel-gium, Nether-lands, Spain, Turkey, UK)	6,195 (4,315 probands with ALS; 1,880 controls)	33/67	Study group: patients with ALS, control group;<br>Whole genome sequence analyses;<br>*VCP* mutation: **inversion** in ***VCP* gene**, associated with ALS<br>*VCP* inversion linked to earlier onset of ALS, but longer patient survival	[11]
**Chinese, East Asian**	169; 4327 East Asians (ExAC data set; [12])	49.7/50.3 for Han Chinese cohort	Study group: Han Chinese patients with familial and early-onset AD;<br>*VCP* genetic variant: common *VCP* **splice variant (rs514492)** associated with AD, identified in South East China and North China cohorts;<br>No association of *VCP* splice variant (rs514492) with AD found for populations with European descent	[13]
**Asian: Japanese**	508	41/59	Study group: Japanese ALS patients;<br>*VCP* genetic variant: **Arg155Cys**, identified for patient with fALS;<br>Clinical manifestations: onset of pathology in upper limb	[14]
**European or European ancestry: Irish**	444	42.3/57.7	Study group: 50 fALS, 394 sALS cases, 311 controls (age-matched, geographically matched);<br>*VCP* genetic variant: **Asn750Ser** (classified as "tolerated" or "benign");<br>Clinical manifestations: bulbar onset of disease	[15]
**Asian: Chinese**	292 (FTD cases)	not specified	Study group: FTD patients in China;<br>VCP mutants in China: **Gly97Glu** (pathogenic); **Thr127Ala** (classified as variant of uncertain significance)<br>*VCP* mutation rate, China: 0.004<br>*VCP* mutation rate, global: 0 – 0.02	[16]
**International**	290	40/60	Study group (literature review): sALS,	[17]

**group** (probands Japanese, Caucasian, Chinese)	(275 sALS, 15 fALS)		fALS<br>79.0% of ALS patients with disease onset in limb, 19.3% with bulbar onset, 1.7% with respiratory onset;<br>VCP-related patients with ALS: 76.1% for Caucasian patients (35/46), 19.6%, for patients of Asian origin (9/46);<br>Pooled analysis: frequency of VCP mutations 0.28% in patients with fALS, 0.06% for sALS;<br>Japanese: frequency of VCP mutations 0.37% for fALS, 0.09% for sALS;<br>Caucasian: frequency of VCP mutations 0.28% for fALS, 0.08% for sALS;<br>Chinese: frequency of VCP mutations 0.08% for fALS, 0.02% for sALS	
**International group** (probands mostly in USA, Europe or Japan)	255	30/70	Study group: cohort with VCP disease;<br>50% of patients with bilateral lower limb weakness at disease onset;<br>clinical symptoms advanced to generalized muscle weakness;<br>PDB: 28.2% of cohort, FTD: 14.3%;<br>*VCP* genetic variants reported: 57;<br>Most frequent variant: **Arg155His**; present in 28% of patients;<br>18 new *VCP* variants identified<br>Correlation between patient origin or ethnicity and *VCP* mutation or disease severity not evaluated.	[18]
**Asian: Chinese**	255	not specified	Study group: Taiwanese patients (Han Chinese) with ALS; 15.3% likely with fALS (FALS), 84.7% with sALS<br>***VCP* genetic variant: no *VCP* mutation reported**	[19]
**International group** (probands in USA, Italy)	210	47/53	Study group: large cohort of fALS cases from unrelated families;<br>VCP mutations present in 1%–2% of studied fALS group;<br>*VCP* genetic variants: **Arg155His**, **Arg159Gly**, **Arg191Gln**, **Asp592Asn**	[20]
**Asian: Chinese**	204	48/52	Study group: unrelated FTD patients with Chinese ancestry<br>*VCP* genetic variant: **Pro188Thr**; *VCP* mutation frequency 0.5%<br>Clinical manifestations: semantic aphasia, expressive disorder	[21]
**European or European**	180	36/64	Study group: Italian fALS cohort; 166 individuals with fALS; 14 with ALS-FTD	[22]

**ancestry: Italian**			**No *VCP* mutation identified as likely cause in fALS cohort of Italian population**	
**Mostly European ancestry: Australian**	179	not specified	Study group: Australian patients, 131 with fALS, 48 with sALS<br>*VCP* genetic variant: synonymous variant c.900C >T in exon 8;<br>Clinical manifestations: not specified for patient carrying the synonymous *VCP* variant	[23]
**Asian: Chinese**	161	33/67	Study group: unrelated patients with ALS, 18.6% with fALS; 81.4% likely sALS;<br>*VCP* genetic variant: **No *VCP* mutation identified in patient cohort**	[24]
**Asian cohort (**details not specified)	152 Asian families; (number of individuals not specified	not specified	Study group: 152 unrelated Asian families with rimmed vacuolar myopathy;<br>Seven individuals with *VCP* mutations;<br>*VCP* genetic variants: **Arg93Cys**, **Arg155Cys**, **Arg155His**, **Arg191Gln, Ala439Pro**,<br>Clinical manifestations: 7 (out of 7) patients with adult-onset slowly progressing muscle weakness, muscle atrophy, changes in skeletal muscle consistent with myopathy and neuropathy, 1 (out of 7) with PDB	[25]
**European or European ancestry: Belgian**	123	50/50 (for 10 patients from two Belgian families)	Study group: 123 FTLD patients and relatives, 157 controls, focus on two Belgian families with *VCP* mutation; one Austrian family with *VCP* Arg159His for comparison<br>*VCP* genetic variants: **Arg159His**;<br>Clinical manifestations: all Belgian patients display FTLD with TDP43 positive inclusions, IBM and PDB with heterogeneous phenotypes	[26]
**Asian: Japanese**	68	40/60	Study group: 68 Japanese individuals from fALS patients; *VCP* mutation identified in one patient;<br>*VCP* genetic variants: **Gly156Cys**;<br>Clinical manifestations: progressive limb muscle weakness, distal limb muscle atrophy	[27]
**Asian: Chinese**	50	62/38	Study of Chinese patients with PDB;<br>**No *VCP* mutation identified in patient cohort**	[28]
**North American cohort: diverse ancestry**	49	49/51	Study group: 49 patients from 9 families; includes patients of German, English, Scottish origin;<br>*VCP* genetic variants: **Arg95Gly**, **Arg155Cys**, **Arg155His**, **Arg155Gln**,	[29]

			**Ala232Glu**;<br>Clinical manifestations: 87% of patients with progressive weakening of proximal muscles, 57% with early-onset PDB, 27% with dementia (mostly FTD)	
**Not specified, possibly French**	~ 36 (not clearly specified)	not specified	Study group: evaluation of members of two families with *VCP* mutations;<br>*VCP* genetic variants: **Arg93Cys**, **Arg155Cys**;<br>Clinical manifestations: FTD for most affected individuals	[30, 31]
**European or European ancestry: Italian**	23	43/57	Study group: 23 patients (15 semantic variant FTD, 8 right temporal variant FTD), 73 healthy age-matched controls;<br>*VCP* genetic variant: **Gly376Glu**, identified in 1 male patient who also carries *TBK1* a I207T mutation;<br>Clinical manifestations: semantic variant FTD	[32]
**Asian: Japanese**	21	43/57	Study group: patients with sporadic inclusion body myositis;<br>**No *VCP* mutation identified in patient cohort**	[33]
**European or European ancestry: French, Spanish**	19 (10 families)	42/58	Study group: VCP myopathy associated with PDB and FTD<br>Eight different *VCP* mutations identified;<br>**Arg155Cys**, **Arg155His**, **Arg159His**, **Arg191Gln**;<br>New mutations identified in study: **Gly157Arg**, **Arg155Ser**, **Pro137Leu**, **Ala439Ser**;<br>Type of mutation does not correlate with disease severity<br>Clinical manifestations: initial clinical symptoms muscle weakness (17 of 19 patients);	[34]
**Asian: Chinese**	16	38/62	Study group: patients of Chinese origin with sporadic juvenile-onset ALS;<br>FUS mutations most frequent genetic cause in patient cohort;<br>**No *VCP* mutation identified in patient cohort**	[35]
**Hispanic**	11	85/15	Study group: members of 5 unrelated Hispanic families with MSP1;<br>*VCP* genetic variant: **Arg159His**;<br>Display atypical phenotype; FTD was the most frequent manifestation, especially in females;	[36]

			Frequencies of the disease manifestations: FTD, 72%; myopathy, 39%; ALS, 8%; PDB, 3%	
**European or European ancestry: Finnish**	9 (members of same family)	44/56	Study group: Finnish family with distal myopathy and *VCP* mutation;<br>Three generations of family evaluated;<br>*VCP* genetic variant: **Pro137Leu**;<br>Clinical manifestation: unusual *VCP* disease phenotype; myopathy only in distal muscles, late-onset dementia, no evidence of PDB	[37]
**Asian: Japanese**	8	25/75	Study group: Japanese patients from five unrelated families;<br>*VCP* genetic variant: **Asp98Val** (novel mutation), **Ile126Phe**, **Arg155Cys**, **Arg191Gln**;<br>Clinical manifestations: IBM or ALS (7/8 patients), demyelinating neuropathy (1/8), ALS-FTD (1/8)	[38]
**European or European ancestry: Swiss**	6 (members of same family)	33/67	Study group: Swiss family with IBM, dementia;<br>Case report; six family members, three generations of the same family;<br>*VCP* mutation: **Ile206Phe**, novel *VCP* mutation located in linker 1;<br>Clinical manifestations: 5 (out of 6 patients) with progressive myopathy; 2 (of 6) with FTD; no reported symptoms consistent with PDB	[39]
**Asian: Chinese**	1	not specified	Study group: Chinese patient with VCP variant Ile206Phe;<br>*VCP* mutation: **Ile206Phe**;<br>Clinical manifestations: myopathy without FTD or PDB (see also Swiss family [39])	[40]
**European or European ancestry: Austrian**	4 (siblings)	75/25	Study group: four siblings of the same family<br>*VCP* mutation: **Arg159His**;<br>Clinical manifestations for females: PDB, then myopathy, no signs of FTD;<br>Clinical manifestations for male: myopathy, then PDB, no signs of FTD	[41]
**Asian; Korean**	3 (siblings)	67/33	Study group: Korean family with MSP1;<br>Three siblings with disease manifestations;<br>*VCP* mutation**: Arg155Cys**;<br>Clinical features of myopathy and/or PDB	[42]
**European or European ancestry: Italian**	3 (siblings)	67/33	Study group: Italian family with early-onset FTD;<br>Three siblings with disease manifestations;<br>*VCP* mutation: **Asp395Ala**;	[43]

			Clinical manifestation: early-onset FTD, behavioral variant frontotemporal dementia (bvFTD) without myopathy or PBD	
**European or European ancestry: Italian**	3	33/67	Study group: Italian family with inclusion-body myopathy and FTD; Three generations of affected family; *VCP* mutation: **Arg155Cys**; Clinical presentation: Two patients with progressive myopathy and dementia; one patient with progressive myopathy and preclinical indicators of PDB	[44]
**African American**	1	100/0	Study group: African American with sALS; *VCP* genetic variant: **Ile151Val**; Clinical presentation: ALS; progressive left lower limb weakness beginning at the age of 68 years; patient death: 70 years	[45]
**Asian; Chinese**	1	100/0	Study group: Chinese female with early-onset PDB; *VCP* genetic variant: **Arg155His**; Clinical presentation: PDB; no myopathy or FTD at disease onset	[46]
**European or European ancestry: German**	1	100/0	Study group: German female patient with inclusion body myopathy and FTD; *VCP* genetic variant: **Arg155Cys**; Clinical presentation:	[47]

Supplemental References

1. Hornbeck PV, Zhang B, Murray B, Kornhauser JM, Latham V, Skrzypek E: **PhosphoSitePlus, 2014: mutations, PTMs and recalibrations.** *Nucleic Acids Res* 2015, **43:**D512-D520.

2. Blum M, Chang H-Y, Chuguransky S, Grego T, Kandasaamy S, Mitchell A, Nuka G, Paysan-Lafosse T, Qureshi M, Raj S, et al: **The InterPro protein families and domains database: 20 years on.** *Nucleic Acids Res* 2020, **49:**D344-D354.

3. Tsitsiridis G, Steinkamp R, Giurgiu M, Brauner B, Fobo G, Frishman G, Montrone C, Ruepp A: **CORUM: the comprehensive resource of mammalian protein complexes–2022.** *Nucleic Acids Res* 2022, **51:**D539-D545.

4. Huang S, Tang D, Wang Y: **Monoubiquitination of Syntaxin 5 Regulates Golgi Membrane Dynamics during the Cell Cycle.** *Dev Cell* 2016, **38:**73-85.

5. Rouillard AD, Gundersen GW, Fernandez NF, Wang Z, Monteiro CD, McDermott MG, Ma'ayan A: **The harmonizome: a collection of processed datasets gathered to serve and mine knowledge about genes and proteins.** *Database* 2016, **2016**.

6. **The Human Protein Atlas** [http://www.proteinatlas.org]

7. Chatr-Aryamontri A, Breitkreutz BJ, Oughtred R, Boucher L, Heinicke S, Chen D, Stark C, Breitkreutz A, Kolas N, O'Donnell L, et al: **The BioGRID interaction database: 2015 update.** *Nucleic Acids Res* 2015, **43:**D470-478.

8. Stelzer G, Rosen N, Plaschkes I, Zimmerman S, Twik M, Fishilevich S, Stein TI, Nudel R, Lieder I, Mazor Y, et al: **The GeneCards Suite: From Gene Data Mining to Disease Genome Sequence Analyses.** *Current Prot Bioinformatics* 2016, **54:**1.30.31-31.30.33.

9. Consortium TU: **UniProt: the Universal Protein Knowledgebase in 2023.** *Nucleic Acids Res* 2022, **51:**D523-D531.

10. Palasca O, Santos A, Stolte C, Gorodkin J, Jensen LJ: **TISSUES 2.0: an integrative web resource on mammalian tissue expression.** *Database* 2018, **2018**.

11. Al Khleifat A, Iacoangeli A, van Vugt J, Bowles H, Moisse M, Zwamborn RAJ, van der Spek RAA, Shatunov A, Cooper-Knock J, Topp S, et al: **Structural variation analysis of 6,500 whole genome sequences in amyotrophic lateral sclerosis.** *NPJ Genomic Medicine* 2022, **7:**8.

12. Lek M, Karczewski KJ, Minikel EV, Samocha KE, Banks E, Fennell T, O'Donnell-Luria AH, Ware JS, Hill AJ, Cummings BB, et al: **Analysis of protein-coding genetic variation in 60,706 humans.** *Nature* 2016, **536:**285-291.

13. Wang G, Zhang DF, Jiang HY, Fan Y, Ma L, Shen Z, Bi R, Xu M, Tan L, Shan B, et al: **Mutation and association analyses of dementia-causal genes in Han Chinese patients with early-onset and familial Alzheimer's disease.** *J Psychiatric Res* 2019, **113:**141-147.

14. Nakamura R, Sone J, Atsuta N, Tohnai G, Watanabe H, Yokoi D, Nakatochi M, Watanabe H, Ito M, Senda J, et al: **Next-generation sequencing of 28 ALS-related genes in a Japanese ALS cohort.** *Neurobiol Aging* 2016, **39:**219.e211-219.e218.

15. Kenna KP, McLaughlin RL, Byrne S, Elamin M, Heverin M, Kenny EM, Cormican P, Morris DW, Donaghy CG, Bradley DG, Hardiman O: **Delineating the genetic heterogeneity of ALS using targeted high-throughput sequencing.** *J Med Genet* 2013, **50:**776-783.

16. Jiang Y, Jiao B, Xiao X, Shen L: **Genetics of frontotemporal dementia in China.** *Amyotroph Lateral Scler Frontotemporal Degener* 2021, **22:**321-335.

17. Feng SY, Lin H, Che CH, Huang HP, Liu CY, Zou ZY: **Phenotype of VCP Mutations in Chinese Amyotrophic Lateral Sclerosis Patients.** *Front Neurol* 2022, **13:**790082.

18. Schiava M, Ikenaga C, Villar-Quiles RN, Caballero-Ávila M, Topf A, Nishino I, Kimonis V, Udd B, Schoser B, Zanoteli E, et al: **Genotype-phenotype correlations in valosin-containing protein disease: a retrospective muticentre study.** *J Neurology, Neurosurgery, Psychiatry* 2022.

19. Tsai PC, Liao YC, Chen PL, Guo YC, Chen YH, Jih KY, Lin KP, Soong BW, Tsai CP, Lee YC: **Investigating CCNF mutations in a Taiwanese cohort with amyotrophic lateral sclerosis.** *Neurobiol Aging* 2018, **62:**243.e241-243.e246.

20. Johnson JO, Mandrioli J, Benatar M, Abramzon Y, Van Deerlin VM, Trojanowski JQ, Gibbs JR, Brunetti M, Gronka S, Wuu J, et al: **Exome sequencing reveals VCP mutations as a cause of familial ALS.** *Neuron* 2010, **68:**857-864.

21. Dong L, Wang J, Liu C, Li J, Mao C, Huang X, Chu S, Peng B, Cui L, Gao J: **Genetic Spectrum and Clinical Heterogeneity of Chinese Frontotemporal Dementia Patients: Data from PUMCH Dementia Cohort.** *J Alzheimer's Disease* 2022, **89:**893-901.

22. Tiloca C, Ratti A, Pensato V, Castucci A, Sorarù G, Del Bo R, Corrado L, Cereda C, D'Ascenzo C, Comi GP, et al: **Mutational analysis of VCP gene in familial amyotrophic lateral sclerosis.** *Neurobiol Aging* 2012, **33:**630.e631-632.

23. Williams KL, Solski JA, Nicholson GA, Blair IP: **Mutation analysis of VCP in familial and sporadic amyotrophic lateral sclerosis.** *Neurobiol Aging* 2012, **33:**1488.e1415-1486.

24. Soong BW, Lin KP, Guo YC, Lin CCK, Tsai PC, Liao YC, Lu YC, Wang SJ, Tsai CP, Lee YC: **Extensive molecular genetic survey of Taiwanese patients with amyotrophic lateral sclerosis.** *Neurobiol Aging* 2014, **35:**2423.e2421-2423.e2426.

25. Shi Z, Hayashi YK, Mitsuhashi S, Goto K, Kaneda D, Choi YC, Toyoda C, Hieda S, Kamiyama T, Sato H, et al: **Characterization of the Asian myopathy patients with VCP mutations.** *Eur J Neurol* 2012, **19:**501-509.

26. van der Zee J, Pirici D, Van Langenhove T, Engelborghs S, Vandenberghe R, Hoffmann M, Pusswald G, Van den Broeck M, Peeters K, Mattheijssens M, et al: **Clinical heterogeneity in 3 unrelated families linked to VCP p.Arg159His.** *Neurology* 2009, **73:**626-632.

27. Naruse H, Ishiura H, Mitsui J, Date H, Takahashi Y, Matsukawa T, Tanaka M, Ishii A, Tamaoka A, Hokkoku K, et al: **Molecular epidemiological study of familial amyotrophic lateral sclerosis in Japanese population by whole-exome sequencing and identification of novel HNRNPA1 mutation.** *Neurobiol Aging* 2018, **61:**255.e259-255.e216.

28. Tao X, Liu L, Yang X, Wei Z, Chen Z, Zhang G, Zhang Z, Yue H: **Clinical Characteristics and Pathogenic Gene Identification in Chinese Patients With Paget's Disease of Bone.** *Front Endocrinol* 2022, **13**.

29. Kimonis VE, Mehta SG, Fulchiero EC, Thomasova D, Pasquali M, Boycott K, Neilan EG, Kartashov A, Forman MS, Tucker S, et al: **Clinical studies in familial VCP myopathy associated with Paget disease of bone and frontotemporal dementia.** *Am J Med Genet Part A* 2008, **146a:**745-757.

30. Ber IL, Martinez M, Campion D, Laquerrière A, Bétard C, Bassez G, Girard C, Saugier-Veber P, Raux G, Sergeant N, et al: **A non-DM1, non-DM2 multisystem myotonic disorder with frontotemporal dementia: phenotype and suggestive mapping of the DM3 locus to chromosome 15q21-24.** *Brain* 2004, **127:**1979-1992.

31. Guyant-Maréchal L, Laquerrière A, Duyckaerts C, Dumanchin C, Bou J, Dugny F, Le Ber I, Frébourg T, Hannequin D, Campion D: **Valosin-containing protein gene mutations: clinical and neuropathologic features.** *Neurology* 2006, **67:**644-651.

32. Rossi G, Salvi E, Mehmeti E, Ricci M, Villa C, Prioni S, Moda F, Di Fede G, Tiraboschi P, Redaelli V, et al: **Semantic and right temporal variant of FTD: Next generation sequencing genetic analysis on a single-center cohort.** *Front Aging Neurosci* 2022, **14:**1085406.

33. Cai H, Yabe I, Sato K, Kano T, Nakamura M, Hozen H, Sasaki H: **Clinical, pathological, and genetic mutation analysis of sporadic inclusion body myositis in Japanese people.** *J Neurology* 2012, **259:**1913-1922.

34. Stojkovic T, Hammouda EH, Richard P, López de Munain A, Ruiz-Martinez J, Camaño Gonzalez P, Laforêt P, Pénisson-Besnier I, Ferrer X, Lacour A, et al: **Clinical outcome in 19 French and Spanish patients with valosin-containing protein myopathy associated with Paget's disease of bone and frontotemporal dementia.** *Neuromuscul Disorders* 2009, **19:**316-323.

35. Zou ZY, Liu MS, Li XG, Cui LY: **Mutations in FUS are the most frequent genetic cause in juvenile sporadic ALS patients of Chinese origin.** *Amyotroph Lateral Scler Frontotemporal Degener* 2016, **17:**249-252.

36. Shmara A, Gibbs L, Mahoney RP, Hurth K, Goodwill VS, Cuber A, Im R, Pizzo DP, Brown J, Laukaitis C, et al: **Prevalence of Frontotemporal Dementia in Females of 5 Hispanic Families With R159H VCP Multisystem Proteinopathy.** *Neurol Genet* 2023, **9:**e200037.

37. Palmio J, Sandell S, Suominen T, Penttilä S, Raheem O, Hackman P, Huovinen S, Haapasalo H, Udd B: **Distinct distal myopathy phenotype caused by VCP gene mutation in a Finnish family.** *Neuromuscul Disorders* 2011, **21:**551-555.

38. Ando T, Nakamura R, Kuru S, Yokoi D, Atsuta N, Koike H, Suzuki M, Hara K, Iguchi Y, Harada Y, et al: **The wide-ranging clinical and genetic features in Japanese families with valosin-containing protein proteinopathy.** *Neurobiol Aging* 2021, **100:**120.e121-120.e126.

39. Peyer AK, Kinter J, Hench J, Frank S, Fuhr P, Thomann S, Fischmann A, Kneifel S, Camaño P, López de Munain A, et al: **Novel valosin containing protein mutation in a Swiss family with hereditary inclusion body myopathy and dementia.** *Neuromuscul Disorders* 2013, **23:**149-154.

40. Wang H, Wu S: **Novel valosin containing protein mutation in a Swiss family with hereditary inclusion body myopathy and dementia.** *Neuromuscul Disorders* 2015, **25:**273.

41. Haubenberger D, Bittner RE, Rauch-Shorny S, Zimprich F, Mannhalter C, Wagner L, Mineva I, Vass K, Auff E, Zimprich A: **Inclusion body myopathy and Paget disease is linked to a novel mutation in the VCP gene.** *Neurology* 2005, **65:**1304-1305.

42. Kim EJ, Park YE, Kim DS, Ahn BY, Kim HS, Chang YH, Kim SJ, Kim HJ, Lee HW, Seeley WW, Kim S: **Inclusion body myopathy with Paget disease of bone and frontotemporal**

dementia linked to VCP p.Arg155Cys in a Korean family. *Arch Neurology* 2011, **68:**787-796.

43. Bruno F, Conidi ME, Puccio G, Frangipane F, Laganà V, Bernardi L, Smirne N, Mirabelli M, Colao R, Curcio S, et al: **A Novel Mutation (D395A) in Valosin-Containing Protein Gene Is Associated With Early Onset Frontotemporal Dementia in an Italian Family.** *Front Genet* 2021, **12:**795029.

44. Gidaro T, Modoni A, Sabatelli M, Tasca G, Broccolini A, Mirabella M: **An Italian family with inclusion-body myopathy and frontotemporal dementia due to mutation in the VCP gene.** *Muscle Nerve* 2008, **37:**111-114.

45. DeJesus-Hernandez M, Desaro P, Johnston A, Ross OA, Wszolek ZK, Ertekin-Taner N, Graff-Radford NR, Rademakers R, Boylan K: **Novel p.Ile151Val mutation in VCP in a patient of African American descent with sporadic ALS.** *Neurology* 2011, **77:**1102-1103.

46. Zhang Y, Gao P, Yan S, Zhang Q, Wang O, Jiang Y, Xing X, Xia W, Li M: **Clinical, Biochemical, Radiological, and Genetic Analyses of a Patient with VCP Gene Variant-Induced Paget's Disease of Bone.** *Calcif Tissue Int* 2022, **110:**518-528.

47. Schröder R, Watts GD, Mehta SG, Evert BO, Broich P, Fliessbach K, Pauls K, Hans VH, Kimonis V, Thal DR: **Mutant valosin-containing protein causes a novel type of frontotemporal dementia.** *Ann Neurology* 2005, **57:**457-461.
